# Interactions of Chromatin Context, Binding Site Sequence Content, and Sequence Evolution in Stress-Induced p53 Occupancy and Transactivation

**DOI:** 10.1371/journal.pgen.1004885

**Published:** 2015-01-08

**Authors:** Dan Su, Xuting Wang, Michelle R. Campbell, Lingyun Song, Alexias Safi, Gregory E. Crawford, Douglas A. Bell

**Affiliations:** 1 Environmental Genomics Group, Laboratory of Molecular Genetics, National Institute of Environmental Health Sciences, National Institutes of Health, Research Triangle Park, North Carolina, United States of America; 2 Institute for Genome Sciences and Policy, Duke University, Durham, North Carolina, United States of America; University of Michigan, United States of America

## Abstract

Cellular stresses activate the tumor suppressor p53 protein leading to selective binding to DNA response elements (REs) and gene transactivation from a large pool of potential p53 REs (p53REs). To elucidate how p53RE sequences and local chromatin context interact to affect p53 binding and gene transactivation, we mapped genome-wide binding localizations of p53 and H3K4me3 in untreated and doxorubicin (DXR)-treated human lymphoblastoid cells. We examined the relationships among p53 occupancy, gene expression, H3K4me3, chromatin accessibility (DNase 1 hypersensitivity, DHS), ENCODE chromatin states, p53RE sequence, and evolutionary conservation. We observed that the inducible expression of p53-regulated genes was associated with the steady-state chromatin status of the cell. Most highly inducible p53-regulated genes were suppressed at baseline and marked by repressive histone modifications or displayed CTCF binding. Comparison of p53RE sequences residing in different chromatin contexts demonstrated that weaker p53REs resided in open promoters, while stronger p53REs were located within enhancers and repressed chromatin. p53 occupancy was strongly correlated with similarity of the target DNA sequences to the p53RE consensus, but surprisingly, inversely correlated with pre-existing nucleosome accessibility (DHS) and evolutionary conservation at the p53RE. Occupancy by p53 of REs that overlapped transposable element (TE) repeats was significantly higher (p<10^−7^) and correlated with stronger p53RE sequences (p<10^−110^) relative to nonTE-associated p53REs, particularly for MLT1H, LTR10B, and Mer61 TEs. However, binding at these elements was generally not associated with transactivation of adjacent genes. Occupied p53REs located in L2-like TEs were unique in displaying highly negative PhyloP scores (predicted fast-evolving) and being associated with altered H3K4me3 and DHS levels. These results underscore the systematic interaction between chromatin status and p53RE context in the induced transactivation response. This p53 regulated response appears to have been tuned via evolutionary processes that may have led to repression and/or utilization of p53REs originating from primate-specific transposon elements.

## Author Summary

It is well established that p53 binds DNA elements near p53 target genes to regulate the response to cellular stress. To assess factors influencing binding to response elements and subsequent gene expression, we have analyzed 2932 p53-occupied response elements (p53REs) in the context of genome-wide chromatin state, DNA accessibility and dynamics, and considered roles for binding-sequence specificity and evolutionary conservation. While p53 occupancy level shows little apparent direct relationship to gene expression change, after grouping expressed genes by their chromatin status at baseline, a relationship between occupancy of p53REs and gene expression change emerged. Analysis of p53RE sequences demonstrated that p53 occupancy was strongly correlated with sequence similarity to p53RE consensus, but surprisingly, was inversely correlated with nucleosome accessibility (DHS) and evolutionary conservation. These data revealed a systematic interaction between p53RE content and chromatin context that affects both quantitative p53 occupancy and the induced transactivation response to exposure. Moreover, this interaction appears to have been tuned via evolutionary events involving transposable elements, which strongly bind p53, but in only a few instances affect gene expression levels. Models of p53-regulated gene expression response that consider both chromatin state and sequence context may prove useful in guiding strategies for cancer prevention or therapy.

## Introduction

Tumor suppressor p53 is activated in response to DNA damage and cellular stress signals and regulates the expression of target genes to elicit cell-growth arrest, DNA damage repair, or apoptosis to prevent the propagation of damaged or compromised cells [1], [2]. Understanding the regulatory logic of p53 is critical to understanding p53 biology in normal and tumor cells. The canonical p53 responsive element (p53RE) is composed of two decamers of RRRCWWGYYY, where R  =  purine, W  =  A or T and Y  =  pyrimidine, separated by a spacer of 0–21 nucleotides (nt), leading to millions of putative p53RE sites in the human genome [3]. About two hundred p53REs have been characterized in detail but p53 chromatin immunoprecipitation sequencing (ChIP-seq) experiments indicate there are thousands of p53 targets and numerous exposure-specific patterns of binding and transactivation [4]–[9]. These patterns have been variously attributed to sequence-specific binding [5], [6], p53 post-translational modifications [10], targeting coactivators/factors [11], post-transcriptional effects [12], as well as, chromatin status at the binding site [13]–[15]. However, the rules governing the sequence specificity and functional output of regulatory interactions between p53 and the genome are not yet fully understood.

A number of *in vitro* studies have clearly demonstrated that p53RE sequence variation, including polymorphisms and spacer length, affect p53 binding to DNA and subsequent transcriptional activation [16]–[20]. These studies show that p53REs with higher similarity to the p53RE consensus display stronger binding and those that contain spacer sequences larger than 1 nt between the decamers show dramatically reduced binding. These studies, however, did not consider the impact of the varied genomic chromatin contexts on p53 binding to response elements and evolutionary conservation of p53REs. In addition, p53 binding motifs occur frequently in primate-specific interspersed repeats, including retroviral long terminal repeats (LTRs [21]), short (SINE [22], [23], such as Alu), and long interspersed nuclear elements (LINEs [24]). Considering the potential role of transposition in the evolution of cis-regulatory elements [25], we ask if these recently evolved p53 binding sites have acquired sequence properties or chromatin contexts which might indicate if they are functionally suppressed or utilized.

Chromatin accessibility can be a primary determinant of transcription factor (TF) occupancy [26], [27]. For example, ligand-activated glucocorticoid receptor binding occurred with near absolute preference (∼95%) for binding sites located in accessible chromatin [26] and recent Encyclopedia of DNA Elements Project (ENCODE) reports [28] have supported this phenomenon for numerous other TFs. In contrast, Nili et. al. [14] described a different situation for the tumor suppressor protein p53, demonstrating that in the tumor cell line MCF7, p53 often binds genomic regions that have high existing nucleosome density and low chromatin accessibility. Other studies have suggested that stress-induced binding at specific *CDKN1A* (p21) p53REs is associated with chromatin structure at these locations [15]. These observations argue for further exploration of the role of chromatin-accessibility in regulating p53 access to DNA regulatory elements. Specific chromatin modifications at histone proteins correlate with TF binding, transcription initiation, elongation, enhancer activity and repression [29]. To elucidate how p53 RE sequences and local chromatin context interact to affect p53 binding and gene transactivation, we have taken advantage of a well-characterized lymphoblastoid cell-culture exposure model system [30]–[32]. The ENCODE project has generated Hidden Markov models (HMM) [32], [33] that annotate the human genome into distinct (15 or 7 HMM states) chromatin states. These models are based on combinatorial patterns of eight informative histone modification marks, Zinc finger CCCTC-binding factor (CTCF), the presence of DNase I hypersensitivity (DHS) and the spatial relationships with gene transcription across nine cell types. The ENCODE ChromHMM model describes the chromatin landscape in unstressed cells and highlights transcriptional regulatory elements reliably and robustly [32], [34]. We have integrated these ENCODE data sets into our experimental data with the aim of deconvoluting the roles for chromatin context/state, p53RE sequence, and p53 occupancy in the transactivation of p53 target genes.

Thus, in the present study we have examined genome-wide changes in p53 binding (ChIP-seq), H3K4me3 (ChIP-seq), gene expression, and DNase I hypersensitivity (DNase-seq) that were induced by the DNA-damaging, chemotherapeutic agent doxorubicin (DXR) in human lymphoblastoid cell lines (LCL). The goal was to assess these changes in the context of ENCODE chromatin states, and consider the role of p53RE sequence content and evolutionary conservation. We revealed several unique relationships, including inverse correlations between p53 occupancy and both chromatin accessibility and evolutionary conservation. Notably, our novel, integrated genome-wide analysis demonstrated that p53RE sequence content is highly correlated with genomic occupancy, and that chromatin state context strongly modulates the relationship between p53 occupancy level and changes in gene expression. Furthermore, the interaction between these features appears to have been shaped by evolutionary selective pressure, likely driven by transposable elements. Our study sheds new light on the complex interrelationship between chromatin state and p53RE sequence in p53 genomic occupancy, and suggests the importance of considering the interactions of sequence content and epigenetic factors in interpreting p53-mediated stress responses.

## Results

### Identification of genomic p53 binding sites and target genes in human lymphoblastoid cells

**Figure 1:**
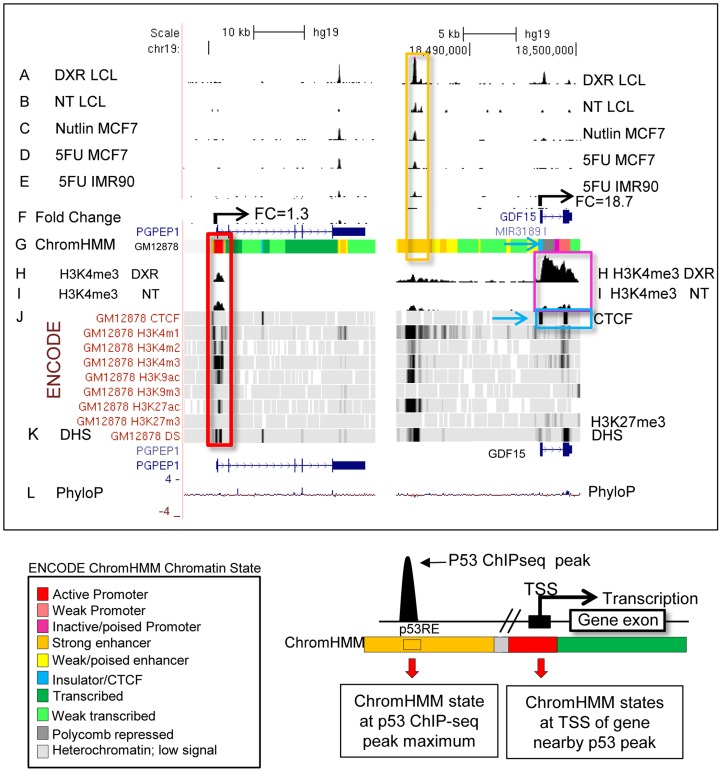
P53 genomic binding relative to chromatin state and histone modifications. Upper Panel: UCSC (University of California Santa Cruz, California, United States) Genome Browser view of experimental data integrated with ENCODE data. Tracks A–B (p53 genomic binding), F (gene expression fold change), H, and I are experimental data for LCLs generated in this study. Track G: ENCODE/Broad chromatin state segmentation (ChromHMM model, unstressed GM12878 cells downloaded from ENCODE). Tracks A–E display p53 ChIP-seq peaks in the *GDF15* region (e.g. orange box). Additional genome-wide information on p53 occupancy was derived from published data as described in methods. Track C: Nutlin–treated MCF7 (breast cancer cell line), Track D: 5-fluorouracil-treated MCF7, Track E: 5-fluorouracil treated IMR90 (embryonic fibroblast). Histone H3 lysine 4 tri-methylation (H3K4me3, tracks H, I) is present at an actively transcribed promoter (PGPEP1, red box) but absent (GDF15, Track I purple box) at the GDF15 promoter displaying insulating CTCF marks (CTCF, track J, blue arrow, blue box. Red box highlights the promoter region of the p53 inducible gene, PGPEP1. Numerous histone modifications align at this position and the ChromHMM track displays red indicative of an active promoter (State 1). In the region within the orange box where p53 peaks were detected, the ChromHMM track displays orange, a high degree of DNase I hypersensitivity (track K, DHS), and multiple histone modifications. Following treatment of GM12878 with DXR, tracks H and I (purple box), change in H3K4me3 marks. Track L shows placental mammalian conservation score (PhyloP). Lower Panel: Illustration of the use of the chromatin state model classification at the p53 ChIPseq maximum (p53RE) or at gene TSS for data analysis in this study.

We carried out p53 activating treatments in LCLs (GM06993, GM11992, and GM12878) using a 0.3 µg/mL dose of DXR and prepared mRNA samples at 4 and 18 hrs of treatment. Chromatin for p53 and H3K4me3 ChIP-seq was prepared from samples collected at 18 hrs, a time point when both growth arrest and apoptosis-related p53 gene targets are occupied [35]. Following DXR-treated p53 ChIP-seq, we detected 6436 peaks at a 5% false discovery rate (FDR) using the QuEST Program [36]. Then, using a cut-off of 30 overlapping sequence reads, we classified 2932 p53 peaks in DXR-treated samples as high-confidence peaks, and with the same sequence read cutoff there were thirty p53 peaks evident under no treatment (NT) conditions. As in primary lymphocytes [37], we observed very limited p53 binding in the absence of stress and the DXR treatment greatly increased the occupancy of p53 at known and newly identified response elements. Among these high-confidence peaks, 91% (2664/2932) have putative p53REs, and 122 of them overlapped known p53 binding sites (Table 1, S1 Fig.). All further analyses focused on these high confidence p53 peaks and the 2415 genes that were associated with them by proximity. Fig. 1 displays a UCSC genome browser view of a sample of these data integrated with ENCODE data from GM12878 cells. Tracks A, B, F, H, I, are experimental data from LCLs generated in our lab for the present study. Tracks A–E display p53 ChIP-seq peaks in the *PGPEP1* (Pyroglutamyl-Peptidase I)*-GDF15* (Growth Differentiation Factor 15) region from this study and two other independent studies [7], [8] using MCF7 cells and IMR90 cells. Many p53 peaks are highly reproducible across multiple experimental conditions (orange box), while other binding locations are specific to treatment and tissue as noted by others [8]. Comparing the present data with three other published p53 ChIP-seq datasets we determined that 69% of 2932 peaks were observed in at least one other independent study. Among the high-confidence peaks unique to this study, 91% (822/903) were observed as significant peaks in an independent p53 ChIP-Seq experiment in LCLs. Histone H3 lysine 4 tri-methylation (H3K4me3, tracks H and I) is present at the *PGPEP1* actively transcribed promoter (red box) but very low at the *GDF15* promoter (track I, purple box), which displays insulating CTCF marks (CTCF, track J, also blue arrows, blue box) in the untreated cells.

**Table 1:** Chromatin state characteristics of some known p53 regulated genes.

p53 Gene	Chromatin State at Occupied p53RE Location	Chromatin State at TSS	H3K4me3 Present at p53RE	CTCF Present	PWM	Mouse-Human % Identity	Fold- Change Gene Expression
PCNA	Open Promoter (State 1)	Open Promoter	+++	−	14.9	0%	1.2
CCNG1	Open Promoter (State 1)	Open Promoter	++	−	18.1	100%	1.2
GADD45A	Open Promoter (State 1)	Open Promoter	++	−	17.5	85%	4.7
P21/CDKN1A	Promoter, Enhancer* (State 3)	Open Promoter	+++	−	17.1	90%	1.9
RRAD	Enhancer (State 3)	Poised	−	+	19.9	65%	7.5
BBC3	Enhancer (State 3)	Open Promoter	+	+	16.3	95%	1.8
GDF15	Enhancer, Promoter*	CTCF Insulated	−	+	19.9	0%	18.7
RND3	Repressed (State 7) Polycomb/Heterochromatin	CTCF Insulated	−/+	+	16.4	80%	5.5
TGFA	Repressed (State 7) Polycomb/Heterochromatin	Poised	−	+	22.0	70%	1.3
PERP	Repressed (State 7) Polycomb/Heterochromatin	Enhancer	−/+	−	12.1	75%	5.3
SULF2	Poised	Poised	−	+	17.1	95%	12.1

* Two large p53 binding peaks, listed relative to size.

To provide a multifaceted view of how the chromatin landscape impacts p53-occupied genes and to explore if p53-inducible genes are actively suppressed at baseline, we have used the ENCODE Hidden Markov Models (two versions, ChromHMM15 [33] and ChromHMM7 Combined [32], which are based on ChIP-seq measurements for multiple histone modifications, CTCF ChIP-seq, DNaseI-Seq, and FAIRE-seq as measured in nine cell lines, including the LCL GM12878, under basal conditions. In Fig. 1, the ChromHMM7 track (G) summarizes the chromatin characteristics of this region of the genome. For example, the red box highlights the promoter region of the p53 inducible gene, *PGPEP1*. Numerous histone modifications and DHS align at this position, and the ChromHMM7 track displays red indicative of an active promoter (State 1). In the same genomic region within the orange box where p53 peaks were detected, the ChromHMM track displays a strong DHS peak and multiple histone modifications (tracks J) particularly H3K27ac, H3K4me1 and H3K4me2 indicative of an active, distal enhancer. We used the ChromHMM7 Combined model (ENCODE website) to classify p53 occupied regions and genes as: 1) active promoter (e.g., *PGPEP1*, active histone marks present: H3K4me2, H3K4me3, H3K27ac, H3K9ac at>93% frequency); 2) promoter flanking region; 3) enhancer (H3K4me1 at>96% frequency); 4) weak enhancer/open chromatin; 5) insulator CTCF enriched; 6) transcribed; 7) repressed, including polycomb and heterochromatin. Some examples of known characterized p53 target genes and their associated HMM states are listed in Table 1.

### Stress-induced H3K4me3 changes correlate with gene expression changes for p53-responsive genes

**Figure 2:**
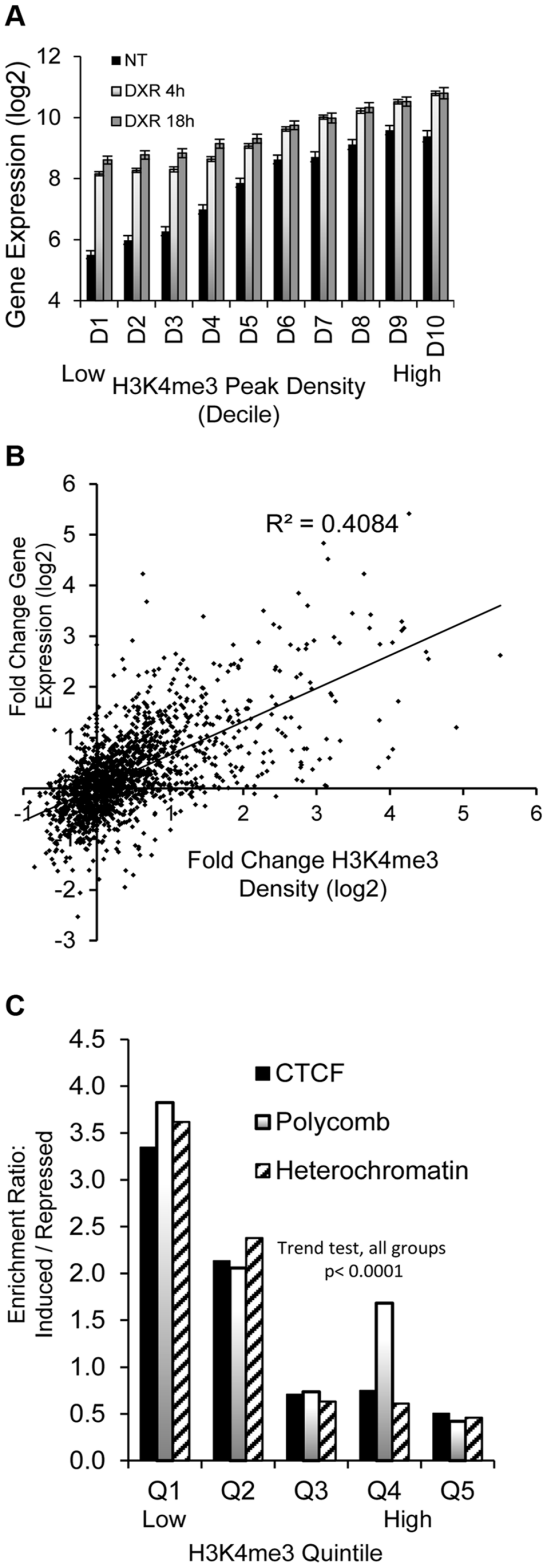
Dynamic change in histone H3K4me3 is correlated with stress-induced gene expression change. (A) Black bars (NT) display linear trend for increasing mean gene expression values with increasing H3K4me3 decile. Grey and open bars show gene expression change is greatest among genes that lack H3K4me3 at baseline. (B) Positive correlation (r^2^ = 0.41, *p* <0.00001) of DXR-induced change in H3K4me3 modification at the TSS with the DXR-induced change in gene expression. (C) Overrepresentation of CTCF, Polycomb, heterochromatic marks among up-regulated genes relative to repressed genes. p53-bound genes were grouped into quintiles based on levels of H3K4me3 at TSS. The frequency of CTCF, polycomb and heterochromatic marks in up-regulated genes relative to their frequencies in down-regulated genes was calculated as enrichment ratio and the enrichment ratio for each quintile is graphed (Y-axis).

The *GDF15* gene had very low expression under no treatment conditions with very little H3K4me3 at its TSS and was strongly induced following p53 activation with the increase of H3K4me3 marks (Fig. 1, purple box). We examined these phenomena across all p53 occupied genes. Of the 2932 DXR-induced p53-occupied regions, 1697 genes had detectable gene expression levels using the Affymetrix Exon Array 1.0 either under no treatment (NT) conditions or after treatment. We grouped expression values into deciles based on baseline H3K4me3 levels at TSS (ordered from low to high, Fig. 2A), we observed a very striking, linear relationship over time (NT, 4 hr, 18 hr after DXR treatment). Expression values for untreated cells (black bars) display a strong linear trend for increasing mean gene expression values with increasing H3K4me3 decile. Following DXR treatment (open bar  =  4 hr, gray bar  =  18 hr), the group of genes with low initial H3K4me3 levels (deciles D1–D3, left side of graph) show the largest change in expression. Thus highly inducible p53-regulated genes typically do not display activating H3K4me3 marks in unstressed cells. We performed regression analysis on the DXR-induced change in H3K4me3 modification with the DXR-induced change in gene expression which showed a significant correlation (Fig. 2B, r^2^ = 0.41, *P*<0.0001). As mentioned, the highly-induced p53 gene *GDF15* displayed both low H3K4me3 and low expression (Fig. 1, purple box) at baseline. However, *GDF15* also displayed insulating CTCF binding (blue box) at TSS. We tested if all low H3K4me3 DXR-induced p53 genes were enriched with repressive chromatin marks at the TSS or gene body relative to down-regulated genes, and determined that highly-inducible p53 genes were characterized by having preexisting chromatin marks indicating insulated (CTCF), polycomb (PcG/H3K27me3), or repressive heterochromatin status (trend test, *P*<0.0001, Fig. 2C). Examples of known p53 genes displaying repressive chromatin marks are listed in Table 1.

### Stress-induced gene expression correlates with steady-state chromatin state (ChromHMM) at TSS

**Figure 3:**
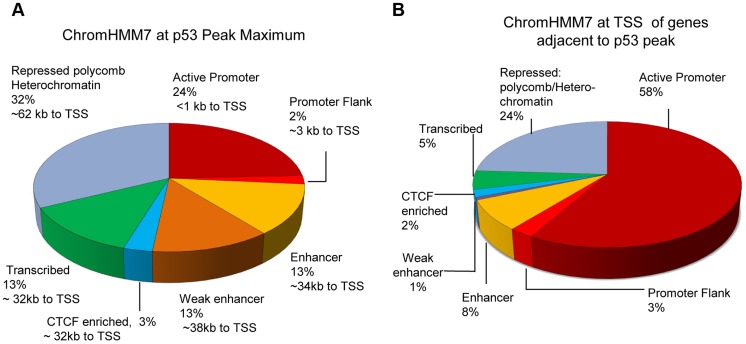
Distribution of p53 peaks among ENCODE combined chromatin states. (A) Distribution of p53 ChIP-seq peaks among chromatin states (ChromHMM7) and the median distance (kb) of the p53-binding locations to TSS of nearby genes. (B) Distribution of chromatin states at TSS of genes nearby p53 peaks.

Fig. 3A demonstrates the distribution of p53 peaks among ChromHMM states and the median distance of the binding locations to the nearest TSS. While the genome of GM12878 cells can be segmented by ChromHMM into promoters (∼1%), enhancers (∼15%), transcribed (∼18%) and repressed regions (∼64%) [33], [38] (S1A Fig.), it is quite clear that p53 binding was highly enriched for promoters, enhancers, and transcribed regions. Most characterized p53RE sites occur in active promoters (S1B Fig.; for examples, see Table 1). While most binding in active promoters was proximal to transcription start sites (TSS), it is notable that 42% of binding locations (those in enhancers, CTCF regions, transcribed regions) have median distances that are at least 32 Kb distal from a TSS, and many binding sites located in repressed regions are much further from a TSS (Fig. 3A). These distal p53 binding locations found in enhancers and repressed chromatin were reproducible in other published p53 ChIP-seq datasets;>76% of these peaks were observed in at least one other experiments (S1 Table).

**Figure 4:**
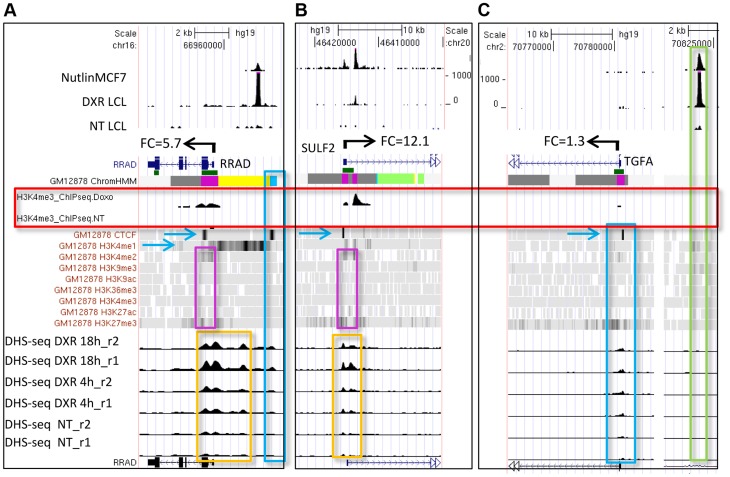
Dynamic changes in chromatin accessibility accompany gene expression change. (A, B) Genes (*RRAD* and *SULF2*) with poised promoters display H3K27me3 and H3K4me2 marks (purple boxes), CTCF (blue arrows), no H3K4me3 marks present at the TSS, and low gene expression levels. DNase I-seq experiments were carried out in duplicate and DHS data for each replicate sample is displayed (lower tracks, orange boxes). Following DXR treatment, H3K4me3 marks appear (red box), mRNA expression increases, and increases in DHS occur at 4 and 18 hrs (orange boxes). (C) *TGFA* with heterochromatin marks at the location of its p53 binding peak (green box). *TGFA* has similar H3K4me2, H3K27me3, and CTCF marks indicative of a poised promoter at baseline and also displays a large p53 peak, however, little change was observed for DHS, H3K4me3 or gene expression.

We also used the ENCODE ChromHMM15 at the TSS to classify the state of the gene nearest to the p53 ChIP-seq peak (illustrated as in Fig. 1 lower panel and Fig. 3B) to identify “poised” promoter regions that carry both repressive (H3K27me3) and activating (H3K4me2/3) marks, and these genes on average showed>3-fold induction (S2B Fig.). Fig. 4A–B displays two genes, *RRAD* (Ras-Related Associated with Diabetes) and *SULF2* (Extracellular Sulfatase 2) with H3K27me3 and H3K4me2 marks (purple boxes), no H3K4me3 marks were present at the TSS, and expression levels were low with no treatment. Following treatment with the DNA damaging agent DXR, H3K4me3 marks appeared (red box) and for each of these poised genes, mRNA expression increased dramatically along with increases in DHS at 4 and 18hrs (orange boxes). However, not all genes follow this pattern. While *TGFA* (Transforming Growth Factor, alpha) (Fig. 4C) had similar H3K4me2, H3K27me3, and CTCF marks indicative of a poised promoter at baseline and also displayed a very large, upstream p53 binding peak, only minimal H3K4me3 and gene expression changes were observed. Thus p53 occupancy had little effect on *TGFA* transactivation, although there was some effect on DHS at the location of CTCF binding (blue box), suggesting other factors may be important for *TGFA* regulation. The patterns observed for chromatin accessibility change (DHS tracks) for individual genes were notable. Peaks of DHS were co-located at sites of H3K4me3 change (red box vs orange box) and also appeared to align with pre-existing H3K4me1/2 and/or CTCF binding patterns (Figs. 4A-C, blue arrows, blue boxes). Among genes that displayed pre-existing heterochromatin marks at the location of their p53 binding peak (e.g. Fig. 4C, *TGFA;* green box), it appeared that p53 binding had minimal detectable effect on nucleosome accessibility (DHS) at some binding sites, even when examined at greater magnification (S3 Fig.).

**Figure 5:**
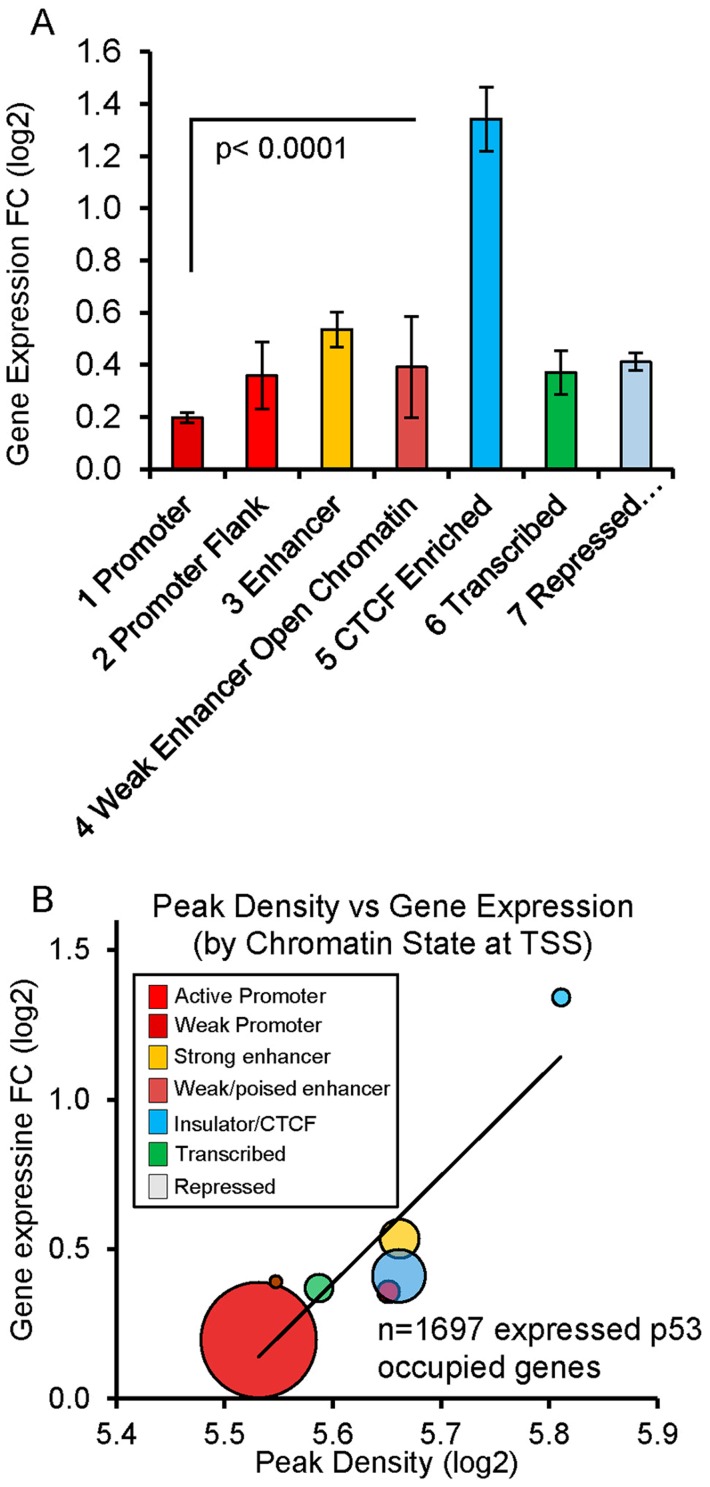
Chromatin state organizes p53 occupancy and gene transactivation. (A) P53-upregulated genes are grouped by ChromHMM at TSS. Genes with CTCF insulation at the TSS prior to treatment were associated with the largest change in gene expression (fold change +/− SEM). Genes displayed in Fig. 4 are examples. (B) Balloon plot depicts the correlation between peak density and change in gene expression after stratifying by ChromHMM state at the TSS. Linear regression was performed on means of the log2 values for each chromatin state and the trend line shown. The size of the circles indicates the relative number of genes in each group.

Comparing the level of p53 occupancy (ChIP-seq peak density) among ChromHMM states for all peaks (n = 2932), we observed significantly higher levels of occupancy for p53 peaks located in distal enhancers relative to promoters and other states (S4A Fig.). However, the result of grouping peaks by ChromHMM at the TSSs of nearby genes indicated that the small group of genes with insulating CTCF at their TSS displayed the highest p53 occupancy (S4B Fig.). The effect of insulated CTCF binding near the TSS prior to treatment was dramatic and this group of genes showed the greatest average change in gene expression (Fig. 5A). Overall p53 peak density displayed a very modest, but significant, correlation with change in gene expression (r^2^  =  0.06, *P* <0.0001, S4C Fig.). However, the more correlated patterns observed for peak density and change in gene expression after stratifying genes by ChromHMM state at the TSS (Figs. 5B, S4D), suggested that chromatin status modifies the relationship between binding/occupancy and transactivation.

### p53 response element sequence content interacts with chromatin context (content versus context)

**Figure 6:**
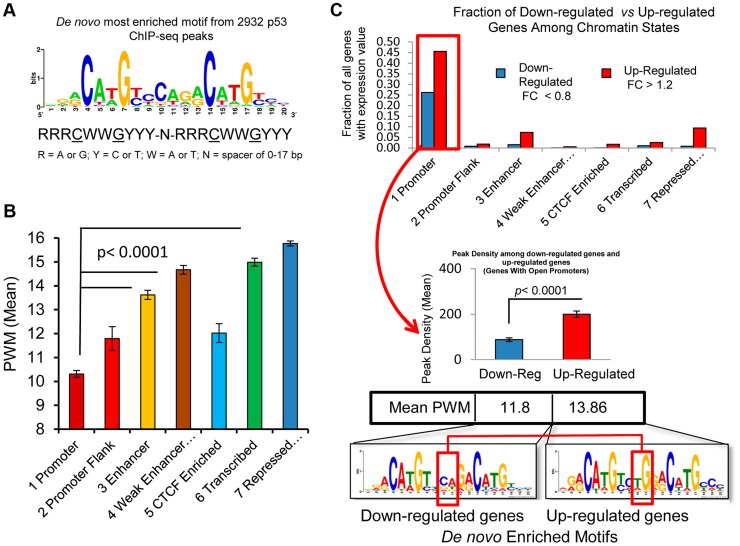
p53 binding occurs at a highly conserved motif and sequence content affect p53 transactivation. (A) Display of the most enriched sequence motif among 2932 peaks identified by *de novo* motif discovery in this study. (B) 2932 p53 peaks were grouped by the chromatin states at the RE. Average PWM scores between chromatin state groups were all significantly different. (C) Distribution of down-regulated and up-regulated genes among ChromHMM states (upper panel). The up-regulated and down-regulated genes were compared for p53 binding, RE Motif and PWM scores (lower panel). Among the genes with open promoters, the p53REs for down-regulated genes have lower average levels of p53 occupancy and PWMs than the up-regulated genes, and display nucleotide differences at the junction of two half-sites in p53RE motifs (red box).

Because recent investigations have suggested that prior chromatin state (before treatment) could predict the occupancy of inducible transcription factor binding [27], [33], [39], we sought to examine the ChromHMM defined state at the location of p53 occupancy and asked the extent to which p53 occupancy level was due to specific p53RE sequence content at the binding site or due to the open-chromatin accessibility (context) at binding locations prior to exposures to DXR. S5A Fig. displays the sequence logo for 103 experimentally verified p53REs that make up the p53 consensus and it closely matches the most enriched motif among p53 ChIP-seq peaks identified by *de novo* motif discovery [40] in this study (Fig. 6A). The most enriched motifs were determined for peaks in each ChromHMM state (S5B–D Fig.) and as expected, each ChromHMM state contained enriched motifs similar to the p53 consensus. However, among peaks lacking full canonical p53REs, p53RE half-sites (decamers) were common (90%, 239/267, Data File S1). The CTCF motif was enriched among peaks associated with down-regulated genes at open promoter state (S5B Fig.). For individual binding sites, we identified putative response elements and considered the calculated binding strengths in relation to chromatin state and other features such as DHS or evolutionary conservation. *GADD45A* (Growth arrest and DNA-damage-inducible, alpha) and *BCL2A1* (BCL2-related protein A1) (S5E–F Fig.) are examples of how these parameters vary considerably across p53REs and other examples are provided in Table 1. We predicted p53RE binding strength using a position weight matrix (PWM) model and a second model based on *in vitro* measurements of p53 binding (Binding PWM, S6A Fig. [16], [31]) and then compared p53RE strength across ChromHMM states (Figs. 6B, S6C). Of interest, mean PWM scores for the p53RE nearest the peak maximum were relatively high in enhancers, consistent with high occupancy at enhancers; the mean PWM scores in repressed chromatin regions were highest. However, the PWMs for the p53REs located in open promoters were much lower. Among p53 peaks associated with genes having open promoters, we observed that both mean PWM and mean peak density were lower among down-regulated genes relative to up-regulated genes (Fig. 6C), and that there was a distinct DNA motif difference as well. Occupancy by p53 was more frequent among induced genes (69% vs 31%; up-regulated genes vs down-regulated genes; Fig. 6C). About 85% (270/317, fold change <0.8) of down-regulated genes displayed active promoters (Fig. 6C) and were highly expressed at baseline (S2A Fig.). Using the functional annotation program DAVID we examined Gene Ontology (GO) annotations of up and down-regulated genes among all chromatin states (Data File S1). The analysis revealed that p53 was the most likely regulator of these genes and that the most significant GO term is “regulation of cell death”. The enrichment for “DNA damage response” and “induction of apoptosis” was particularly strong when we compared highly occupied p53REs vs less occupied p53REs (Data File S1, DAVID analysis).

The p53 consensus RE is also characterized by a variable spacer region of 1–21 nt between the two decamer half-sites, and since the PWM is calculated independently of the variable spacer, these represent independent features. The length of the p53RE spacer is known to reduce the affinity of p53 for binding to its consensus sequence and transactivation [41]. While spacers longer than 3 nt were relatively rare, the weak p53REs that were located in active promoters were more likely to have spacers and have larger spacers (S6B Fig.), which was associated with weaker binding sequences (Fig. 6B). The negatively correlated patterns of spacer length and PWM strength (S6B–D Fig.) were further evidence that these weak p53REs may be functional. Thus, p53REs located in active promoters were weaker by two distinct criteria, while elements in more distant locations in enhancers and in repressed chromatin states appear to be significantly stronger.

### Negative correlation of both p53RE PWM score and occupancy with chromatin accessibility (DNase 1 HS)

**Figure 7:**
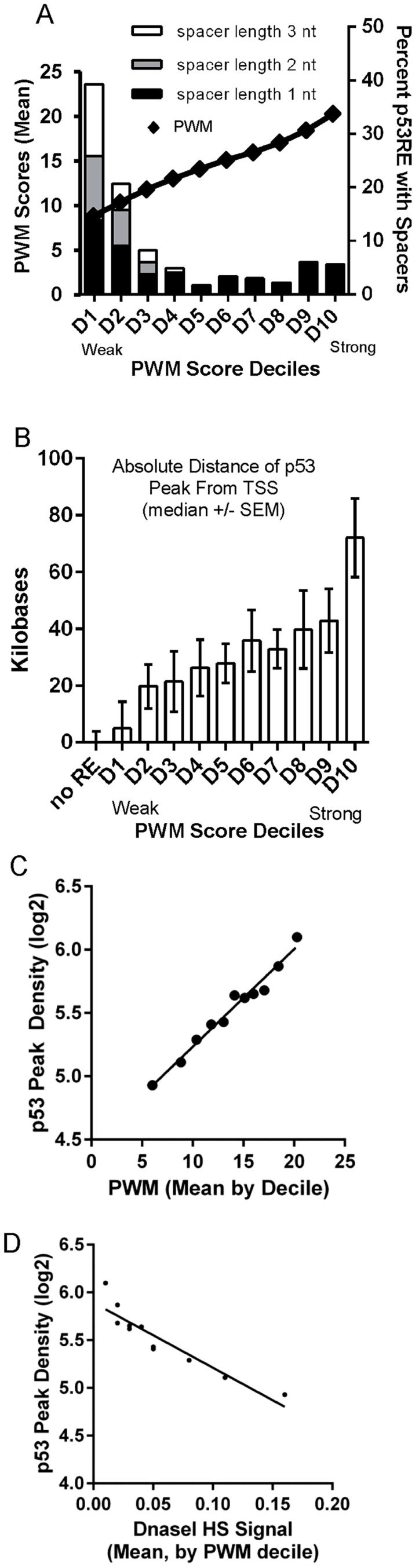
Best PWMs are correlated with chromatin accessibility and p53 occupancy. (A) For each p53 occupied region, the best PWM is the one closest to the position with maximal p53 binding signal. The best p53RE PWM scores are ordered from low to high, and grouped into deciles. The mean value of p53RE PWM scores for each decile is displayed as diamond symbols on the line graph (left X axis). Stacked bars represent frequency of spacers in each p53RE decile (right Y axis). P53REs with weak, small PWM scores more frequently contain spacers between half-sites. (B) P53 peaks containing the strongest PWM scores are distal to the TSS (decile D10). (C) Regression of average peak density versus average best PWM of REs grouped by PWM deciles. (D) Regression of average p53 peak density against average DHS signal at REs under NT conditions grouped by PWM deciles. Peaks containing the weakest PWMs have lower occupancy and are associated with the highest DHS scores.

To directly visualize the relationship between p53RE binding strength score (PWM), occupancy, and chromatin accessibility, we ordered the dataset by computed PWM scores from low to high, and grouped data into deciles (Fig. 7). We first tested the relationship between PWM values and the frequency of spacers between the half-sites (Fig. 7A, spacers of various lengths shown by stacked bars) and observed spacers to be more common in p53REs with weaker PWM scores (consistent with S6B, S6D Figs.). A surprising result was that p53 peaks containing the strongest PWM scores proved to be far distant from TSSs (Fig. 7B, decile D10) while the weakest p53REs were <1 kb from the TSS. Particularly notable was the linear relationship between p53RE binding strength (PWM score) and p53 occupancy (peak density, Fig. 7C). To assess if this observation was unique only to DXR-treated LCLs, we accessed p53 ChIP-seq data from 5-fluorouracil (5FU) treatment of MCF7 breast cancer epithelial cells [8], calculated p53RE PWM scores within p53 ChIP-seq peaks (S7A Fig.), and compared PWM with ChIP-seq density (S7B Fig.). A similar linear relationship between PWM score and peak density was observed in MCF7 cells. By comparing these results with other published p53 ChIP-seq datasets [7]–[9] we determined that p53 ChIP-seq peak density was also highly significantly correlated with reproducibility of p53 peaks across multiple experimental systems (Data File S1). ChIP-seq peaks containing the weakest p53RE PWMs had lower occupancy and were associated with the highest DHS scores in untreated cells (Fig. 7D). This observation was also confirmed in MCF7 cells (S7C Fig.). Thus we observe that open promoters that were characterized by the presence of pre-existing accessible chromatin (DHS) were permissive for p53 binding to the weakest elements. Conversely, the p53 binding sites with the highest PWMs and highest occupancy tend to be in the most distal p53 binding locations (∼60 kb from a TSS and in heterochromatin), most of these sites display very low DHS, and these observations were independent of cell type.

### Evolutionary conservation at p53 binding sites varies with chromatin state and RE binding strength, and is influenced by the presence of transposable elements

**Figure 8:**
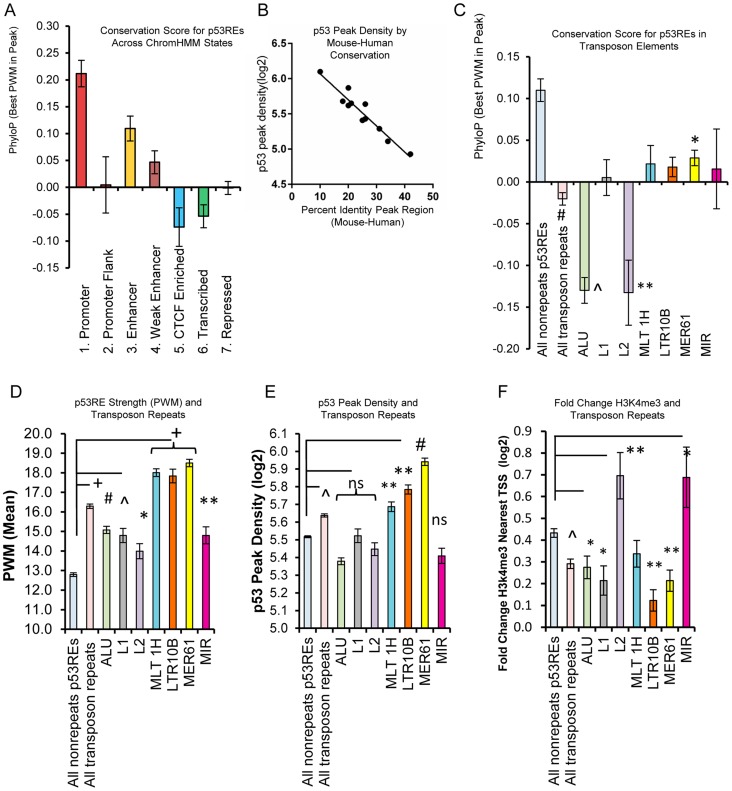
p53REs show diverse levels of conservation, occupancy, strength, and transactivation across chromatin states and among families of transposable elements. (A) p53REs were grouped by ChromHMM and an average PhyloP score (placental mammals) across the 20nts of the p53RE was calculated. Values are plotted as averages for each ChromHMM group. (B) p53REs were ordered by their PWM value into deciles, and p53 peak density was regressed against human-mouse conservation score, displaying an inverse correlation between peak density and human-mouse conservation. (C) Mean PhyloP conservation scores differ among p53REs embedded in different TE families. (D) Mean PWM scores for p53REs embedded in different TE families were significantly higher than nonTE p53REs. (E) p53 occupancy was significantly higher for p53REs embedded in MLT1H, LTR10B and MER61 families. (F) Mean H3K4me3 fold change at nearby gene TSSs were significantly less among most TEs but higher for L2 and MIR families. * p <5×10^−2^; ** p <1×10^−2^; ∧p <1×10^−4^; ^#^ p <1×10^−12^, ^+^ p <1×10^−27^.

Gene regulatory regions, including promoters and enhancers typically display high evolutionary conservation, presumably due to negative selection pressure to preserve functional DNA elements [42]–[44]. However, we have previously noted that evolutionary conservation varied greatly among well-characterized functional p53REs, with some human elements displaying little homology with other mammals [45], and we wondered if p53RE conservation varies by chromatin state. Grouping p53REs by their ChromHMM state and plotting their average PhyloP score across the 20nts of the RE dramatically demonstrates this phenomenon (Fig. 8A). Response elements located in promoters and enhancers were highly conserved relative to those in CTCF enriched, transcribed, or repressed regions. When we ordered p53REs by PWM decile we revealed an inverse correlation between p53 occupancy and evolutionary conservation of the peak region, either by mouse-human percent identity (Fig. 8B) or by PhyloP score (S8A Fig.). These analyses demonstrate that more highly conserved p53REs are relatively weak p53REs and display lower occupancy relative to poorly conserved sequences.

Some primate-specific transposable elements (TEs) are known to contain p53REs [21], [22], [24]. To evaluate their influence on p53RE conservation scores, we analyzed the overlap of occupied p53REs (full sites with spacer ≤3) with repeat elements categorized in Repeat Masker (http://www.repeatmasker.org/faq.html). We observed that 33% (970 of 2932) of p53-occupied sites were embedded in distinct families of repeat elements, with a significant enrichment for the endogenous retroviral elements MER61, LTR10B1, and MLT1H, as well as others (Data File S1). Most TE-associated p53 ChIP-seq peaks (82.7%; 803/970) were also observed in independent experimental datasets (Data File S1). The presence of TE-associated p53REs strongly affected conservation scores among HMM states (Figs. 8A, S8B–C). For example, TE frequency was inversely associated with PhyloP scores, being high in heterochromatin, intermediate in enhancers, low in promoters (S8B, C Fig.). As expected, average PhyloP values were significantly lower among all TE-associated p53REs relative to non-TE p53REs (Fig. 8C). Surprisingly, when we examined PhyloP values for individual families of TEs, we observed Alu and L2 family p53REs were dramatically negative compared to all others (Fig. 8C). We considered if chronological age of the TE might explain this pattern but this is not the case because Alu repeats are among the oldest of TE families while L2 is among the youngest [46]. Negative PhyloP values are predicted to be fast evolving and may be related to recent evolutionary selection as suggested by Pollard, et al [47] and this would be consistent with the p53 pathway acquiring new primate specific functions.

To test if TE-associated p53REs affect sequence content (PWM, Fig. 8D) and occupancy (p53 ChIP peak density, Fig. 8E), we compared TE-associated p53REs with that of non-repeat p53REs. We observed significantly higher PWM scores in all TE-associated p53REs, with several families particularly high (MER61, MLT1H, LTR10B; Fig. 8D) and the p53 ChIP-seq peak density plot displays a similar, correlated pattern (Fig. 8E). Thus the relationship between PWM and ChIP-seq peak density that we observed previously among all p53 peaks (Fig. 7C), holds among families of repeats (S8D Fig.). If these peaks were functionally associated with nearby genes we would expect treatment-induced changes in H3K4me3 peak density at the TSS. Fig. 8F shows that genes nearby TE-associated p53REs display less average H3K4me3 changes than nonTE-associated genes. Notable exceptions to this phenomenon include genes nearby L2 (66 genes) and MIR families (46 genes) of TEs which showed significantly higher levels of DXR-induced gene expression (Fig. 8F). Two known p53 genes (*DDR1*, p53RE in L2a; *EPHA2*, p53RE in MIRb) are among these groups. The p53REs that overlap L2 family repeats seemed unusual in that they show both strongly negative PhyloP values (Fig. 8C) and display changes in H3K4me3 at nearby TSS locations (Fig. 8F). We explored several of these L2-associated p53 binding sites in more detail and browser views of two strongly negative PhyloP p53REs that also show altered H3K4me3 at the TSS following p53 activation by DXR are shown in S9A–B Figs. (H3K4me3 data in Data File S2). In addition, four L2-associated p53REs with negative PhyloP values that only show DHS changes at the location of the binding site are given in S9C–F Fig.

## Discussion

We assessed genome-wide *in vivo* p53 occupancy, H3K4me3, DHS and gene expression in human lymphoblastoid cells treated with the DNA-damaging chemotherapeutic agent DXR. Previous studies of functional regulatory variation indicate that LCLs are responsive to a variety of treatments [28], including p53-activating, DNA-damaging agents like DXR [16], [31] and ionizing radiation [48]. On a genome-wide scale, we quantified the relationship between dynamic, stress-induced changes in gene expression and changes in H3K4me3 (Figs. 2A–B, 4A–B), a mark present on the nucleosomes flanking nucleosome-free regions that coincide with active TSSs [49], [50]. Notably, the group of genes with the greatest change in expression appear to be actively repressed at baseline (Figs. 2C, 4A–B), either via CTCF insulation, polycomb marks or the presence of heterochromatin. Strikingly, when we stratify the 1697 genes that display mRNA expression by the ChromHMM state at their TSS, we observe a linear trend between patterns of quantitative p53 occupancy (peak density) and fold change gene expression (Fig. 5B). Based on single-gene experimental models of p53 occupancy and transactivation, this relationship is not unexpected, but the clarity achieved by considering chromatin state on a genome-wide scale is dramatic (comparing S4C Fig. with Fig. 5B). Thus, chromatin state models created under steady-state conditions, such as the ENCODE ChromHMM, were highly predictive of genome-wide p53-mediated responses, and can help elucidate the mechanisms that enable those responses. This interesting finding should be examined in other inducible gene expression pathways and other cell types.

While greater than 50% of well-studied p53-regulated genes have binding sites proximal to a TSS, only about 25% of p53 binding was near a TSS in our study (Fig. 3B). Binding sites located in distal enhancer elements (∼34-kb from a TSS) comprised 26% of peaks, while another ∼30% were located in regions of heterochromatin even more distal to a TSS (median of 70 kb from a TSS). Approximately 80% of these distal sites in repressed chromatin were also identified in other genome-wide studies of p53 binding [6]–[9]; however, most of these sites remain functionally uncharacterized. Studies utilizing long distance chromatin capture techniques (HiC, 3C) under p53-activated conditions will be needed to confirm distal interactions between p53 binding and transactivation. Nevertheless, we did observe a clear relationship between p53 occupancy of p53REs located in repressed chromatin regions, and inducible transactivation of many genes within repressed regions (e.g., *TGFA*, *ANK1, GDNF*, *CYP4F3*).

In our study 91% (2664/2932) of the high confidence p53 occupied regions contained sequences similar to the motif created from 103 known p53 binding sites (S5A Fig.). We asked if p53 binding sequences located in different chromatin functional regions might display characteristic differences, and we observed that among the 592 peaks occurring in active promoters, the most prominent motif closely matched the p53 consensus (S5B Fig., p = 3.9×10^−63^). Interestingly, the next most common motif in peaks located in active promoters closely resembled the CTCF motif (p = 4.8×10^−27^) and this motif was present more commonly in down-regulated genes. However, these p53-related CTCF motifs were not associated with the CTCF insulator function as these regions displayed high nucleosome accessibility and the genes had high average expression levels. In contrast, genes classified as CTCF insulated by the ENCODE HMM model had low basal expression level but high inducibility (Fig. 5A–B). Among the most highly induced genes, we observed that prior to treatment, there was enrichment of repressive chromatin features such as CTCF, polycomb (PcG-H3K27me3) or heterochromatin (Fig. 2C). Genes with poised promoters carrying both H3K27me3 and H3K4me2 at the TSS (Figs. 4A–C, S2C) were an important group among these inducible genes and include a number of known p53 response genes (*RRAD, TGFA, PERP, GPR87, RNF144B, TNFSF10A*).

Espinosa and Gomes [13], [51] have hypothesized that loss of CTCF insulation activity could allow for inducible expression of some poised p53 genes. DXR induced changes in DHS that co-locate precisely with CTCF binding in untreated cells are consistent with this notion. Akdemir et. al. [52] proposed that changes in H3K27me3 status at poised genes via a mechanism of p53-JMJD3/UTX interaction could drive the conversion of poised genes to actively expressed genes. However, in the present experiment, the expression of these chromatin remodeling factors were not detectable. We did observe that changes in H3K4me3 were strongly confluent with altered DHS patterns and consistent with transcriptional activation of some poised p53 genes like *SULF2* following treatment. The detailed mechanism drives the release of these specific genes from their poised state is an area of future study. Down-regulated genes had quite different characteristics; most down-regulated genes (85%) had open promoters (Fig. 6C) and displayed high expression at baseline (S2A Fig.). Interestingly, both the density of p53 binding, and the calculated p53RE PWM score associated with down-regulated genes were significantly lower compared with p53REs from induced genes (*p*<0.0001, Fig. 6C). Nikulenkov et. al. [8] observed a similar lower peak density among down-regulated genes. Wang et. al. [19] analyzed a small number of p53REs and suggested there was a preferred motif for down-regulated genes that is different from up-regulated genes. We also detected differences in the most enriched motif between the two groups of sequences. Specifically at nucleotides 10 and 11 in the p53RE motif, “TG” was present in the up-regulated REs and a “CA” motif occurred in the down-regulated genes (Fig. 6C). Consistent with this altered motif, the p53REs in down-regulated genes have a weaker match with the canonical sequence and lower mean PWM (Fig. 6C). However, this change is unlikely to be a general feature of p53REs because among all chromatin states many up-regulated genes contain the “CA” motif at nucleotides 10, 11. Pathway and functional analysis of each chromatin state by DAVID analysis showed that p53 is the most likely regulator of these genes and that the most significant GO terms include “regulation of cell death” and “response to DNA damage stimulus”.

The relative importance of DNA sequence content and chromatin landscape in determining a transcription factor's genomic binding has been subjected to considerable experimental exploration [28], [53]–[55], including several with p53 [14]–[16], [56]. Chromatin accessibility determines the binding of glucocorticoid hormone receptor [26] and *STAT1* [57]. However, in the present study, we detected that a large fraction (29%) of p53 binding occurred at distal, repressed chromatin regions. The quantitative level of p53 occupancy was high in these regions of high intrinsic nucleosome occupancy, which is consistent with the findings of Nili et. al [14]. Thus, p53 occupancy and inducible gene expression occurred in regions of high nucleosome density indicated by existing H3K27me3 marks, heterochromatin, or low DHS. Notably, at the level of individual binding sites (e.g., upstream of *TGFA*), high levels of p53 occupancy can occur with minimal local displacement of nucleosomes (Figs. 4C, S3; as detected by DHS-seq). However, for poised genes marked with both H3K27me3 and H3K4me2 (Fig. 4A–C), p53 occupancy was associated with nucleosome accessibility changes at the TSS, but not necessarily at the p53 binding site. In these cases, as gene expression was induced, we observed the open DHS region spreading from the TSS, and the DHS pattern resembles the DXR-induced H3K4me3 pattern.

A particularly notable result is the relationship between quantitative occupancy and binding strength of the p53RE sequence relative to consensus. Modeling p53RE binding strength using a statistical approach (PWM), or with an experimental binding-based model (binding PWM, S6A Fig.), we observed genome-wide correlations between binding strength and occupancy (peak density). This relationship was further validated using p53 ChIP-seq data generated from a different laboratory using a different cell type and treatment (S7A–C Fig.). Thus, p53RE sequence content strongly affects occupancy which is in agreement with several *in vitro* studies that reported single nucleotide changes in the p53RE sequence or the spacer length alter p53 binding, transactivation of constructs or gene expression [16], [20], [41]. Recently a single nucleotide variation in a p53RE in the *KITLG* gene was demonstrated to alter p53 binding, transactivation of *KITLG* and be strongly associated with cancer [58]. However, our present study is the first genome-wide study to demonstrate the quantitative relationship between sequence content and genome-wide occupancy levels.

Surprisingly, p53RE PWM scores and occupancy were strongly negatively associated with chromatin accessibility and evolutionary conservation (see Figs. 7D, 8B, S7C) at the binding site. That is, more conserved, relatively weak p53REs were observed to be found in accessible chromatin regions. Conversely, many strong p53REs with low conservation scores were located in heterochromatic regions with very low DHS. The p53REs located within enhancers displayed relatively high levels of conservation, PWM score, occupancy and accessibility. The contrast in levels of conservation and chromatin accessibility among p53REs is driven by the presence of primate-specific repeat elements of viral transposon origin that are characterized by low conservation, high PWM, high occupancy but low accessibility. This high occupancy, low accessibility and minimal nucleosome displacement observation is consistent with the Cui et. al [22] hypothesis that p53REs are accessible on the external surface of nucleosomes. Cui et. al. described this for Alu-associated p53REs; however, our data suggests that it may be a general feature of p53-occupied REs in heterochromatin, particularly those in retroviral-associated TEs (e.g. MLT, LTR, MER). Over many millions of years of evolution transposable elements have rearranged primate genomes [46], and the presence of a multitude of p53 binding sites in various TEs [21]–[24] likely is related to an evolutionary mechanism for creating p53REs from TE sequences. Our present study uniquely reveals the interrelationships between TEs, p53RE sequence content, occupancy, and chromatin state.

There are several interesting and important questions concerning the functional nature of these TE-associated p53REs. Are these TEs actively suppressed (as a genome defense) and can they be reactivated in tumors or in normal cells following genomic stress like DNA damage? Leonova et. al. suggest that TEs are epigenetically suppressed and p53 is involved in transcriptional silencing of them [59]. We observed that TE-associated p53REs were significantly stronger REs and were more highly occupied than non-repeat p53REs; however, most reside in repressive, closed chromatin. In addition, this occupancy did not typically translate into induced H3K4me3 levels at nearby gene TSSs or changes in gene expression. This would be consistent with suppression of these TE-associated p53REs by p53 binding. Also, one may ask, what are the characteristics of p53REs located in TEs that would allow them to be co-opted (exaptation) and used to regulate the expression of nearby genes [60], [61]? It is unknown which features of chromatin or changes in chromatin structure might facilitate the utilization or exaptation of transposon-derived p53REs (e.g., domestication of transposons). While this question cannot be easily answered with the present data, it is intriguing that we observe some p53REs that overlap with TE-repeats (Alu and L2) display strongly negative PhyloP values suggesting the possibility of recent evolutionary selection [47]. In addition, if we consider that p53 occupancy of TE-associated p53REs is sometimes accompanied by altered DHS, H3K4me3 or gene expression at nearby genes (as shown in S9 Fig.), it seems likely that many of them are functionally utilized.

Thus it is possible that both suppression and exaptation have occurred with many strong, newly-evolved TE-associated p53REs being actively suppressed via chromatin state but a small number of them are exapted and brought into the p53 regulatory network for use under stress conditions. These observations imply a potential functional link between the variation in genomic p53RE sequence, chromatin state and gene regulation, which has co-evolved over time. As pointed out by Leonova et. al. [59], characterizing the p53-mediated deregulation of transposable elements in tumors could be useful in clinical cancer diagnoses. Understanding how all of these variables interact and impact the p53-regulated response to DNA damage in primary and tumor cells will be important both for developing strategies for prevention of cancer in healthy individuals and for understanding outcomes in cytotoxic therapy for cancer.

## Materials and Methods

### Cell culture

HapMap (http://hapmap.ncbi.nlm.nih.gov/) CEU human lymphoblastoid cell lines GM06993, GM11992, and GM12878 (Coriell Cell Repositories, Camden, NJ) were cultured in RPMI 1640 medium supplemented with 15% heat-inactivated fetal bovine serum (Life Technologies) and 1X antibiotics/antimycotics (Life Technologies). Cells were incubated at 37°C with 5% CO_2._ During experiments, cells were grown in petri dishes and treated with 0.3 µg/ml doxorubicin (Sigma, St. Louis, MO) for ChIP-seq, DNase I hypersensitivity, and gene expression.

### Gene expression microarray analysis

GM12878 cells were cultured in triplicate and were either untreated or treated with 0.3 µg/ml doxorubicin for 4 and 18 hrs. Total RNAs was extracted using total RNA Miniprep kit (QIAGEN) with DNase 1 digestion. Total RNA was subjected to the standard cDNA synthesis, dye labeling and hybridization as per Affymetrix's protocols for the Human Exon ST 1.0 array (Affymetrix, Santa Clara, CA), and processed at the NIEHS Microarray Core. Exon expression data was analyzed through Affymetrix Expression Console using gene-level RMA summarization and sketch-quantile normalization methods. The *t*-test was used to calculate the significance of gene expression change. All the Affymetrix microarray data used here have been deposited into the GEO database (GSE51709). DXR induced gene expression array data was validated in a separate cell line using the Illumina gene expression array platform (Data File S1), as well as RT-PCR for selected genes. For replication by qRT-PCR, the cDNA was generated using the First-Strand Synthesis system (Life Technologies). For each biological replicate, target genes were amplified in triplicate using FAM probes designed to span exon junctions for each target gene and PCR Master Mix (Life Technologies). Studies investigating the relationship between gene expression in lymphoblast cell lines and *in vitro* p53 binding have been reported previously ([16], [31]).

### Chromatin Immunoprecipitation-Sequencing (ChIP-Seq)

Lymphoblastoid cells were seeded in 15-cm dishes and treated with 0.3 µg/mL doxorubicin or vehicle water for 18 hrs. Chromatin from 100×10^6^ cells was used for each p53 ChIP-seq experiment. ChIP for p53 was carried out essentially as described in the Agilent Mammalian ChIP-on Chip protocol Version 20.0 (Agilent, Santa Clara, CA) using mouse monoclonal antibody DO-7 (BD Biosciences, San Jose, CA) or a non-specific rabbit IgG conjugated to secondary Dynal magnetic beads (Life Technologies). H3K4me3 ChIP was carried out with the H3K4me3 antibody (EMD Millipore #07-473, Billerica, MA). ChIP and input DNA were quantified using Qubit (Invitrogen). NIH Intramural Sequencing Center (NISC, Bethesda, MD) and the National Center for Genome Resources (NCGR, Santa Fe, NM) created the libraries using the standard Illumina ChIP-seq protocol. ChIP or input libraries were sequenced using Illumina Genome Analyzer IIx. For validation of occupancy, p53 ChIP-PCR was performed in triplicate using Sybr primers designed to span p53RE peaks in promoters of p53 target genes on a 7900 HT real-time PCR machine (Applied Biosystems).

### DNase I hypersensitivity mapping

Genome-wide mapping of DNase I-hypersensitive sites (DHS) was carried out on 2 replicates of untreated and DXR-treated GM12878 cells for 4 and 18 hrs. For DHS profiling, intact nuclei were treated with DNase I, and the DNase I-hypersensitive fraction was analyzed by sequencing as previously described [62]. Data from two biological replicates under each experimental condition are displayed as tracks in the UCSC genome browser in Figs. 4, S3, S9. For analysis of DHS relative to ENCODE genome wide chromatin state, we downloaded ENCODE/OpenChrom (Duke University, Durham, NC) GM12878 DHS data (Fig. 1, track K) [28], [33]. These data (DHS signal intensity) were averaged across p53 binding regions, transformed (log2 +1), grouped by PWM decile and plotted versus p53 peak density (Figs. 7D and S7C).

### p53 ChIP-seq, and H3K4me3 ChIP-seq data analysis

p53 ChIP-seq, H3K4me3 ChIP-seq, and input DNA sequence reads generated from the Illumina GAIIx were aligned against the human reference sequence (GRCh37p5, or hg19, June 2011) using the Burrows-Wheeler Alignment (BWA) Tool (Li et al., 2008). The uniquely mapped short reads were used to identify regions of the genome with significant enrichment in p53-associated DNA sequences. The peak detection was performed by QuEST 2.4 software [36] using the ‘Transcription factor binding site’ setting (bandwidth of 30 bp, region size of 300 bp) or the “Histone-type ChIP” setting (bandwidth of 100 bp, region size of 1000 bp) for H3K4me3 ChIP-seq, and the ‘stringent peak calling’ parameters (corresponding to 50-fold ChIP to input enrichment for seeding the regions and 3-fold ChIP enrichment for extending the regions).

We also used three published studies of p53 ChIP-seq from Nutlin-treated MCF7 cells [8], 5FU-treated MCF7 cells [8], 5FU-treated IMR90 cells [7], Nutlin and DXR-treated Human osteosarcoma U2OS cells [9], which deposited the raw reads at the NCBI SRA database. All sequence reads were aligned against the human reference sequence (GRCh37p5, or hg19, June 2011) using the Burrows-Wheeler Alignment (BWA) Tool (Li et al., 2008). For these published studies the peak detection was also performed by QuEST 2.4 software [36] using the ‘Transcription factor binding site’ setting (bandwidth of 30 bp, region size of 300 bp) for p53 ChIP-seq and the ‘stringent peak calling’ parameters. In Fig. 1 our H3K4me3 ChIP-seq tracks were prepared using the MACS program in order to display comparability with ENCODE tracks.

### *De novo* motif discovery

MEME Suite [40] was used for the *de novo* motif analysis of the genomic DNA sequences identified by ChIP-seq experiments. Briefly, repeat-masked DNA sequence for each peak was trimmed to 100 bp, centered to the maximal signal of original peak. The MEME algorithm was then applied to identify the top 10 most enriched motifs with a length from 6 to 25.

### Searching for putative p53REs using the position weight matrix

Position weight matrix (PWM) score was used to computationally estimate the binding strength to p53. The p53 PWM model was built on 103 published, experimentally validated p53RE sequences from the literature (NCBI PUBMED database), by converting nucleotide frequency values to a position weight matrix score as described [63]. Next the PWM score for any putative p53RE was calculated by summing the individual matrix values that correspond to the observed nucleotide at each position in that site. The potential p53REs in a genomic sequence were detected by sliding a window along the input sequence, considering the spacer in p53REs [64]. Briefly, at each chromosomal position, PWM score calculations were performed on DNA sequences with 20–23 nucleotides, corresponding to p53REs with a spacer of 0, 1, 2, and 3, respectively. For DNA sequences longer than 20, the first 10 nucleotides and the last 10 nucleotides were concatenated first to make a sequence of 20 nucleotides. For fast searching, a pattern composed of at least 3 of 4 core C and G nucleotides in the p53RE consensus was applied before PWM calculation. In addition, the same calculations were performed on the reversed complimentary sequences. In total, at each chromosomal position, eight calculations were performed. At a given location a putative p53RE was reported based on the following three criteria: (i) the sequence matches the search pattern; and (ii) the PWM score is above the PWM score threshold; and (iii) it contains the shortest spacer and has the largest PWM score. A second computational analysis based on measured binding in an *in vitro* binding assay, termed a binding PWM, was determined using a method described in Noureddine et. al. [31].

### Analyses of ENCODE ChIP-Seq Datasets and ChromHMM state for GM12878 cells

Datasets and sample information for histone modifications and CTCF binding for the GM12878 cell line were from the ENCODE project (http://genomes.ucsc.edu) via the laboratory of Bradley Bernstein (Broad Institute) [34], at the Massachusetts General Hospital/Harvard Medical School. The chromatin state segmentation was produced in Manolis Kellis's Computational Biology group at the Massachusetts Institute of Technology [33], [54]. We created analysis files based on 2932 p53 ChIP-seq peaks that had≥30 overlapping sequence reads (Data File S1) and 2415 genes associated with these peaks (many genes had multiple peaks, Data File S2). We merged ENCODE data (ChromHMM15 and ChromHMM7 Combined models, PhyloP, DHS) into our data analysis files that included ChIP-seq peak density (p53, H3K4me3), gene expression (intensity at each time point, fold change). Examples of these data are displayed in Fig. 1. In the various analyses we grouped p53 peaks (or genes) based on the H3K4me3 status of nearby genes, the ChromHMM7 Combined status at p53 peaks or at the TSS of nearby genes, or on the computationally calculated binding strength (PWM). ChromHMM7 Combined model failed to call states for some regions and the ChromHMM15 was used in these cases. ChromHMM15 states 14 and 15 (repetitive) were dropped from analysis as no p53 ChIPseq peaks were uniquely mapped to these regions. We also specifically looked at expression levels of genes with “poised” promoters carrying H3K4me1/2 marks and H3K27me3 marks (ChromHMM15 state 3). To visualize linear trends related to H3K4me3 or PWM scores we used decile analysis in which PWM values were ordered from low to high and grouped into deciles. We then evaluated other parameters relative to these linear trends. Regression, *t*-tests, and other statistics were calculated using Graphpad Prism (GraphPad Software).

### Evolutionary conservation analysis

To evaluate the evolution constraint on p53 binding sites, we utilized the placental mammal basewise conservation scores (PhyloP, phylogenetic p-values) downloaded from the UCSC genome browser website. These scores are computed from the PHAST package (http://compgen.bscb.cornell.edu/phast/) for multiple alignments of 45 vertebrate genomes to the human genome. The phyloP scores measure acceleration (faster evolution than expected under neutral drift) as well as conservation (slower than expected evolution). Sites predicted to be conserved are assigned positive scores, while sites predicted to be fast-evolving are assigned negative scores. The absolute values of the scores represent log p-values under a null hypothesis of neutral evolution. The sequence alignment and human-mouse conservation score were analyzed as previously described [45]. Transposon element repeats cataloged by Repeat Masker were accessed at UCSC genome browser website and aligned with p53REs to determine overlap.

### Data access

Sequence reads for ChIP-seq experiments are deposited in the NCBI SRA database (p53 GSE46991, H3K4me3 GSE51713) and the NCBI Gene Expression Omnibus (GEO: http://www.ncbi.nlm.nih.gov/geo). Gene expression data are available at GEO under accession number GSE51709.

## Supporting Information

S1 Fig Genome-wide chromatin states relative to p53 binding regions. (A) The general distribution of chromatin states across the genome (similar ChromHMM states are condensed here) (Ernst et al, [33]) (B) Distribution of 122 known p53RE sites detected in this study among ChromHMM states.
(TIF)

S2 Fig Chromatin states affect gene expression change of p53-occupied genes. (A) Log2 gene expression for p53-occupied genes among different chromatin states after DXR-treatment for 4 and 18 hrs. The p53-associated genes are grouped by ChromHMM state at their TSS and the average log2 gene expression levels are shown. Left panel, genes with down-regulation (FC<0.8) after DXR treatment. Right panel, genes with up-regulation (FC>1.2) after DXR treatment. (B) Genes with “poised” promoter regions that carry both H3K27me3 and H3K4me2 marks at TSS as identified by ChromHMM15 displayed large average changes in gene expression following DXR treatment. The average of log2 gene expression at 4 and 18 hrs after DXR treatment are indicated as bars (mean+/−SEM). (C) Frequencies of repressive marks present at genes after grouping genes by quintiles based on the H3K4me3 levels from low to high. Higher frequency of repressive CTCF, polycomb and heterochromatin marks was observed among the genes with low H3K4me3 levels (Q1) as compared to genes with high H3K4me3 levels (Q5).
(TIF)

S3 Fig Binding of p53 to TGFA region occurs with minimal displacement of nucleosomes, while *ANK1* displays modest change in displacement. Dynamic chromatin accessibility (DHS) at the p53 binding sites for *TGFA* and *ANK1* are examined at greater magnification. DHS vertical scale expanded 10-fold relative to Fig. 4A–C. Bottom track displays the peaks and gaps in the input DNA sequence that approximate inter-nucleosomal and nucleosome-protected DNA (147-nt). Purple box is about 150-bp wide.
(TIF)

S4 Fig Peak density correlates with gene expression changes. (A) p53 ChIP-seq peaks were grouped by ChromHMM state at the peak maximum and average peak density (mean+/−SEM) is graphed. Difference in peak density between peaks in promoters and enhancers was significant (student t-test). (B) Comparison of p53 peak density (log2) after grouping peaks by the chromatin state at the TSS of the nearby genes (N = 2415). (C) Regression plot of p53 peak density (log2) and fold change gene expression (log2) for 2415 p53-associated genes. (D) HMM chromatin state organizes p53 binding and gene expression. Balloon plot is used to display fold change gene expression as a function of p53 peak density after grouping values by chromatin state at the TSS of the nearby genes (N = 2415). The p53 peak density was derived from the averaged p53 ChIP-seq peak density of three DXR-treated LCL cell lines. The size of the points indicates the relative number of genes in each group.
(TIF)

S5 Fig De novo motif analysis of p53 peaks in different chromatin states. The most enriched motifs were determined for each ChromHMM state by MEME. (A) Sequence logos for consensus p53 RE generated from 103 known p53 REs. (B) Sequence logos for the peaks located in active promoters closely matched the p53 consensus (motif 1) and the CTCF motif (motif 2). Peaks associated with down-regulated genes displayed CTCF motifs. (C) Several motifs recovered from peaks located in enhancers each resembled variations on the p53 consensus. (D) Sequence logos for the peaks located in heterochromatin closely matched the p53 consensus. (E) The *GADD45A* p53 binding site is in an open region that is well conserved with mouse. (F) In contrast the *BCL2A1* peak is>20 kb upstream, shows no conservation with mouse, and is located in heterochromatin. The *BCL2A1* p53RE (F) has a very high PWM, displays higher occupancy and matches the p53RE consensus better than *GADD45A* p53RE (E).
(TIF)

S6 Fig Sequence content (PWM) as organizing principle of genomic occupancy. (A) Correlation between the computed PWM and the experimentally verified binding PWM after grouping genes by PWM score deciles. (B) Left Panel, comparison of mean length of RE spacers after stratifying genes by their chromatin states at REs. Right Panel, frequency of individual spacer lengths among chromatin states. (C) Comparison of the sum of PWMs of all p53REs under each p53 peak by chromatin state at peaks. Weaker p53REs that are located in active promoters also have larger spacers. (D) Genes were grouped by ChromHMM at p53 peaks, the mean p53RE PWM score and spacer length of p53REs was computed and regressed. *In vitro* validation of p53 binding to 29 p53REs shown to be occupied in this experiment was reported in Noureddine et. al [31].
(TIF)

S7 Fig Sequence content (PWM) in relationship to p53 binding and chromatin accessibility in MCF7 cells. (A) p53 peaks in 5-FU-treated MCF7 cells are grouped based on PWM scores of REs into 10 deciles from low-to high; no RE peaks are in decile D1. (B) Linear relationship between PWM score and peak density is observed for p53 binding in MCF7 cells. (C) Inverse relationship between DHS and peak density was observed in MCF7 cells after stratifying genes by PWM deciles. Data from Nikulenkov et al.[8].
(TIF)

S8 Fig Relationships between p53 peak density, RE conservation score and PWM scores for repeats family-associated p53 RE. (A) Relationship between patterns of peak density and evolutionary conservation (PhyloP). (B) Counts of p53REs embedded in representative families of transposon elements. (C) The percentage of repeats-associated p53RE in each chromatin state. (D) Regression of p53 peak density and PWM scores for each family of repeat elements.
(TIF)

S9 Fig Altered DNaseI hypersensitivity at L2 transposable element associated p53 binding sites following DXR treatment. (A–B) These genes show both DNaseI change at p53RE and also H3K4me3 change at the TSS. (C–F). These genes show DNaseI change at the site of p53 binding in the TE. ChromHMM is shown in upper track, followed by DXR p53 ChIP-seq peaks, gene model, RepeatMasker tracks, PhyloP, and DNaseI hypersensitivity Seq (NT, 4 hrs DXR, 18 hrs DXR, 2 replicates each, indicated by r1, r2).
(TIF)

S1 Table Distribution of Features of High Confidence p53 Peaks and p53REs Among ChromHMM7 States.
(TIF)

S1 Data File Su p53 2932 peaks, HMM7 at peak, PWMs, phyloP, multiples, repeats, DAVID.xlsx.
(XLSX)

S2 Data File Su 2415 p53 genes, HMM7 at TSS expression, H3K4me3 FC, TE repeats 10_31_14.xlsx.
(XLSX)
