# Supplementary figures and images for: Interactions of Chromatin Context, Binding Site Sequence Content, and Sequence Evolution in Stress-Induced p53 Occupancy and Transactivation
Source: PLoS Genet. 2015 Jan 8;11(1):e1004885. doi: 10.1371/journal.pgen.1004885 (PMC4287438; doi:10.1371/journal.pgen.1004885)

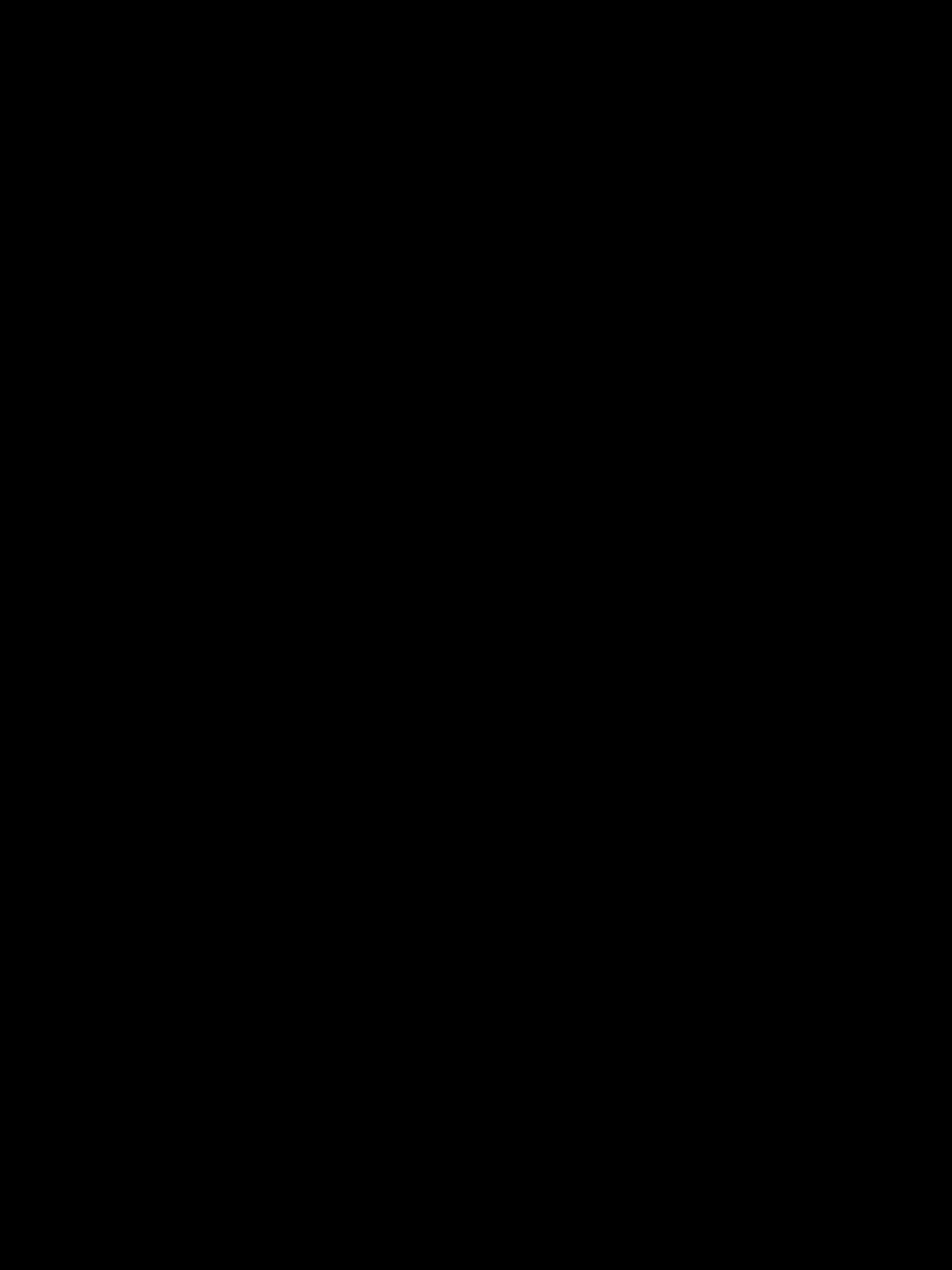

Supplement: S1 Fig — Genome-wide chromatin states relative to p53 binding regions. (A) The general distribution of chromatin states across the genome (similar ChromHMM states are condensed here) (Ernst et al, [33]) (B) Distribution of 122 known p53RE sites detected in this study among ChromHMM states. (TIF) [file pgen.1004885.s001.tif]

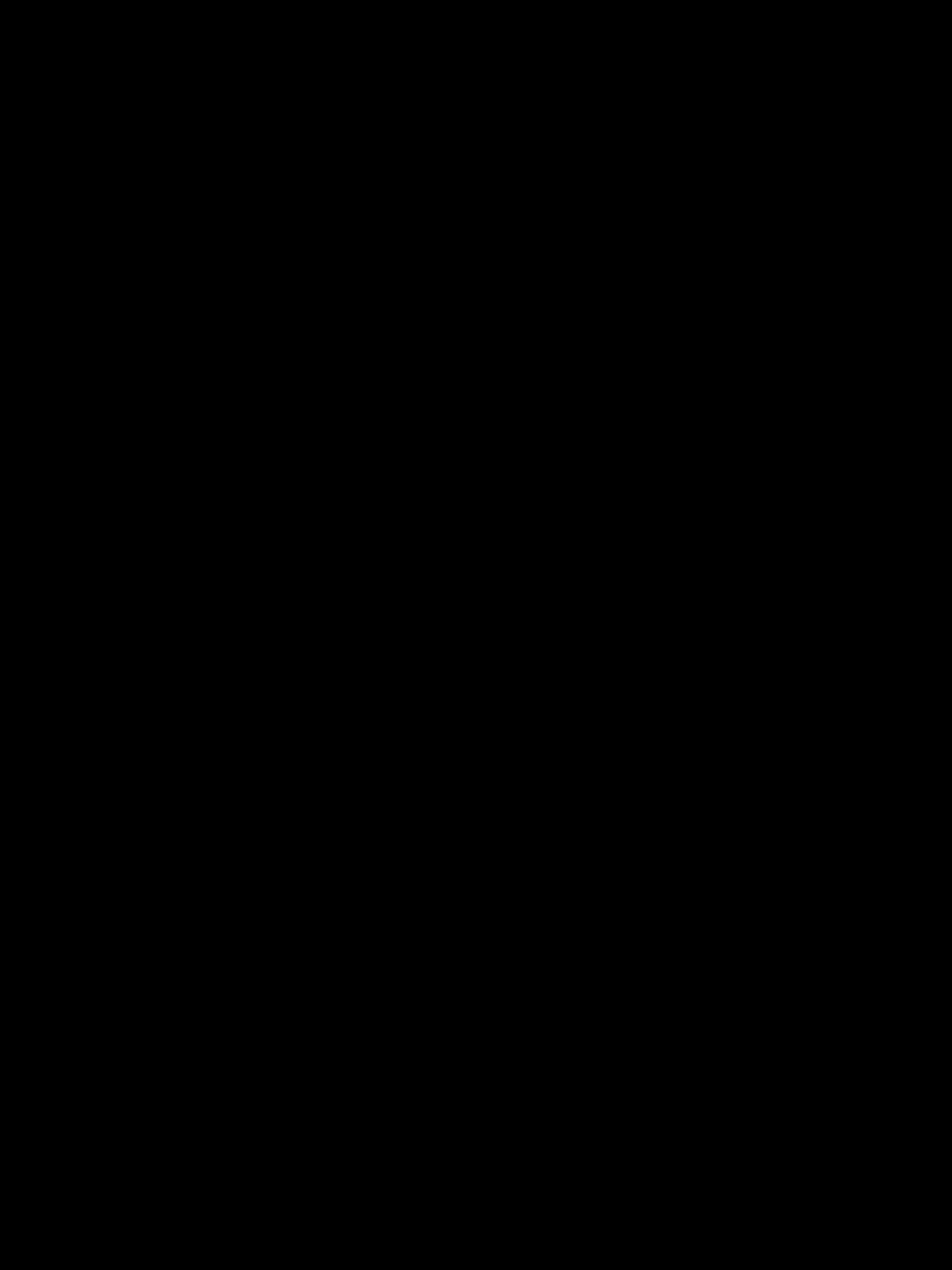

Supplement: S2 Fig — Chromatin states affect gene expression change of p53-occupied genes. (A) Log2 gene expression for p53-occupied genes among different chromatin states after DXR-treatment for 4 and 18 hrs. The p53-associated genes are grouped by ChromHMM state at their TSS and the average log2 gene expression levels are shown. Left panel, genes with down-regulation (FC<0.8) after DXR treatment. Right panel, genes with up-regulation (FC>1.2) after DXR treatment. (B) Genes with “poised” promoter regions that carry both H3K27me3 and H3K4me2 marks at TSS as identified by ChromHMM15 displayed large average changes in gene expression following DXR treatment. The average of log2 gene expression at 4 and 18 hrs after DXR treatment are indicated as bars (mean+/−SEM). (C) Frequencies of repressive marks present at genes after grouping genes by quintiles based on the H3K4me3 levels from low to high. Higher frequency of repressive CTCF, polycomb and heterochromatin marks was observed among the genes with low H3K4me3 levels (Q1) as compared to genes with high H3K4me3 levels (Q5). (TIF) [file pgen.1004885.s002.tif]

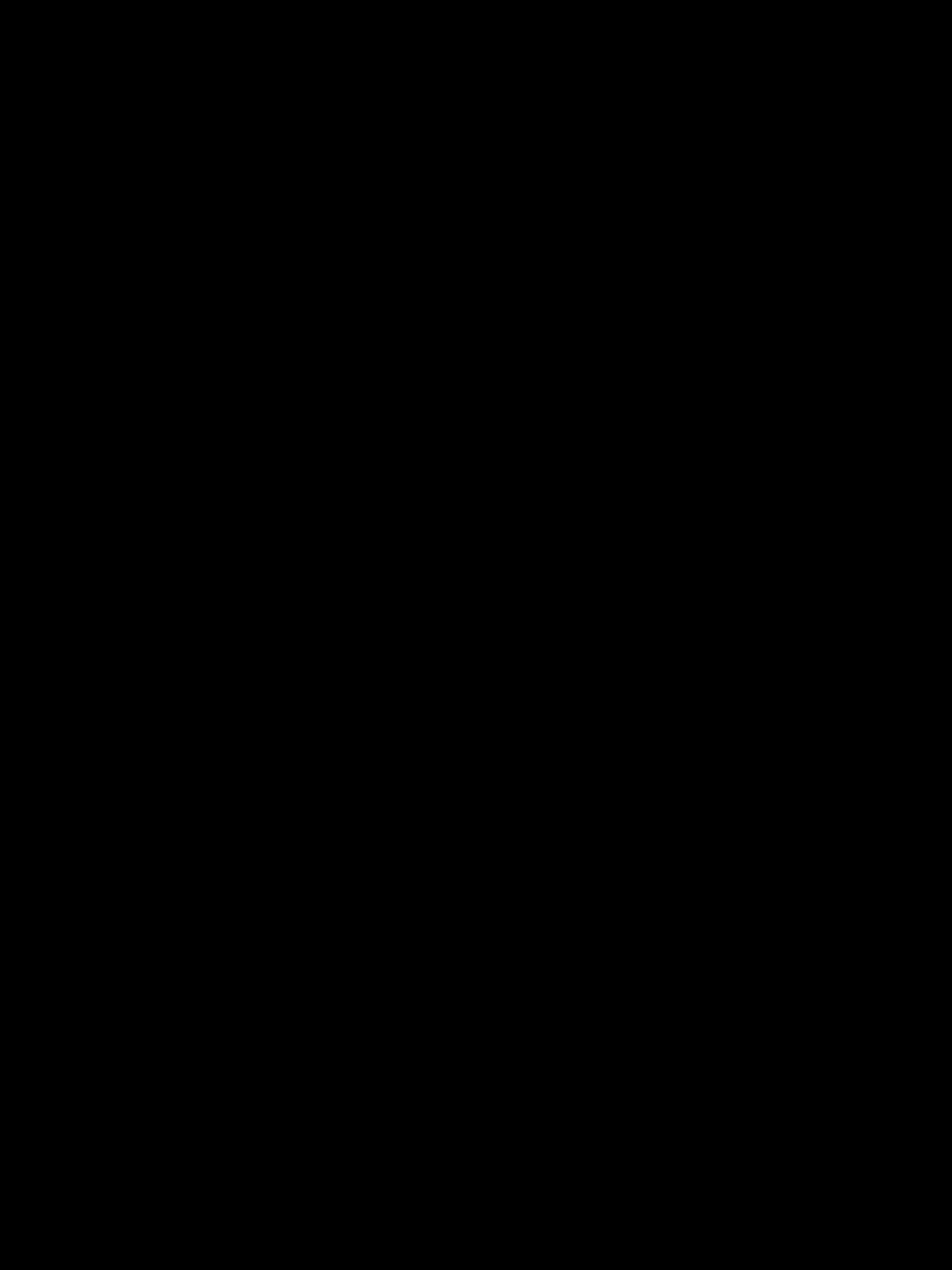

Supplement: S3 Fig — Binding of p53 to TGFA region occurs with minimal displacement of nucleosomes, while ANK1 displays modest change in displacement. Dynamic chromatin accessibility (DHS) at the p53 binding sites for TGFA and ANK1 are examined at greater magnification. DHS vertical scale expanded 10-fold relative to Fig. 4A–C. Bottom track displays the peaks and gaps in the input DNA sequence that approximate inter-nucleosomal and nucleosome-protected DNA (147-nt). Purple box is about 150-bp wide. (TIF) [file pgen.1004885.s003.tif]

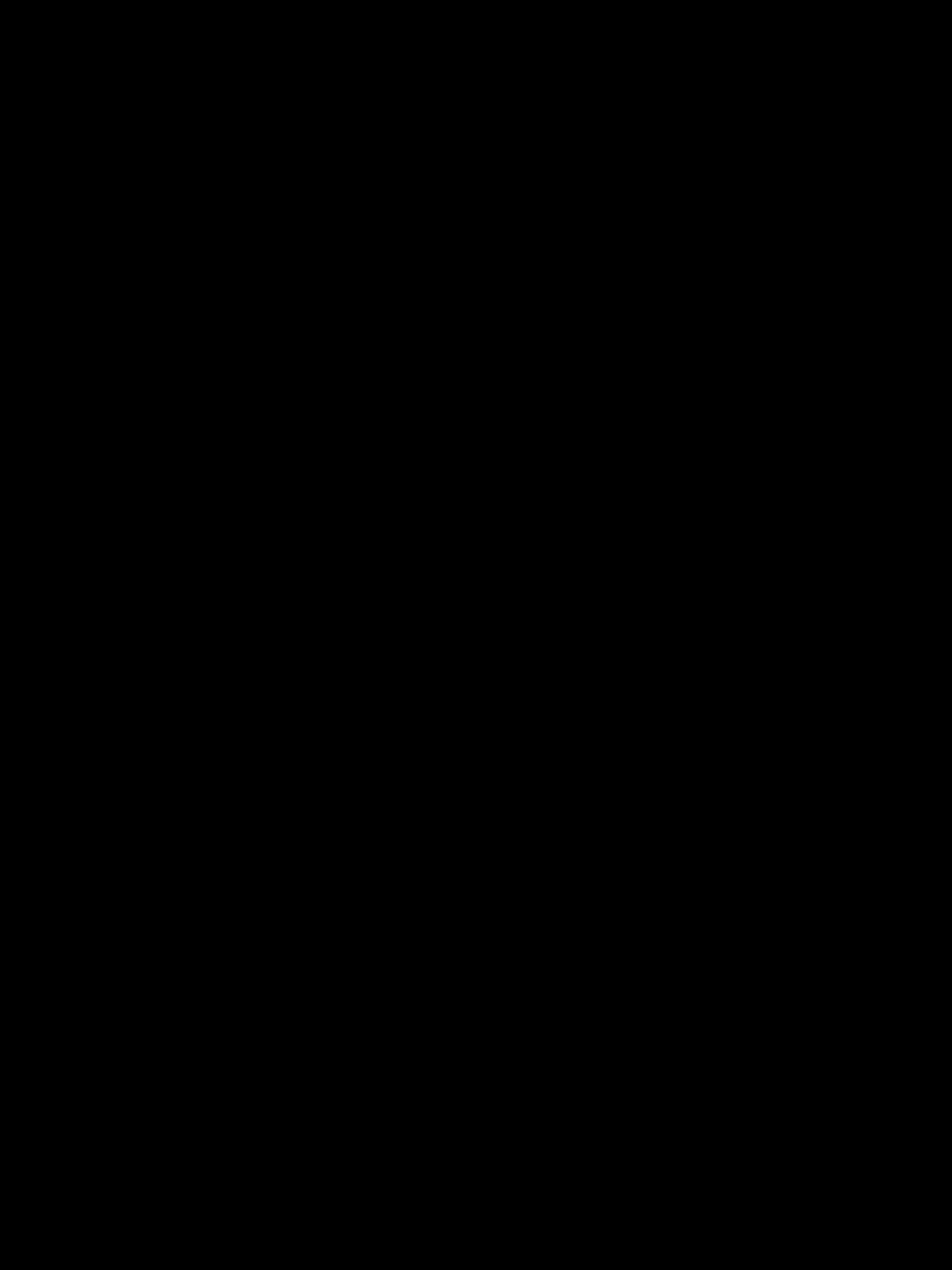

Supplement: S4 Fig — Peak density correlates with gene expression changes. (A) p53 ChIP-seq peaks were grouped by ChromHMM state at the peak maximum and average peak density (mean+/−SEM) is graphed. Difference in peak density between peaks in promoters and enhancers was significant (student t-test). (B) Comparison of p53 peak density (log2) after grouping peaks by the chromatin state at the TSS of the nearby genes (N = 2415). (C) Regression plot of p53 peak density (log2) and fold change gene expression (log2) for 2415 p53-associated genes. (D) HMM chromatin state organizes p53 binding and gene expression. Balloon plot is used to display fold change gene expression as a function of p53 peak density after grouping values by chromatin state at the TSS of the nearby genes (N = 2415). The p53 peak density was derived from the averaged p53 ChIP-seq peak density of three DXR-treated LCL cell lines. The size of the points indicates the relative number of genes in each group. (TIF) [file pgen.1004885.s004.tif]

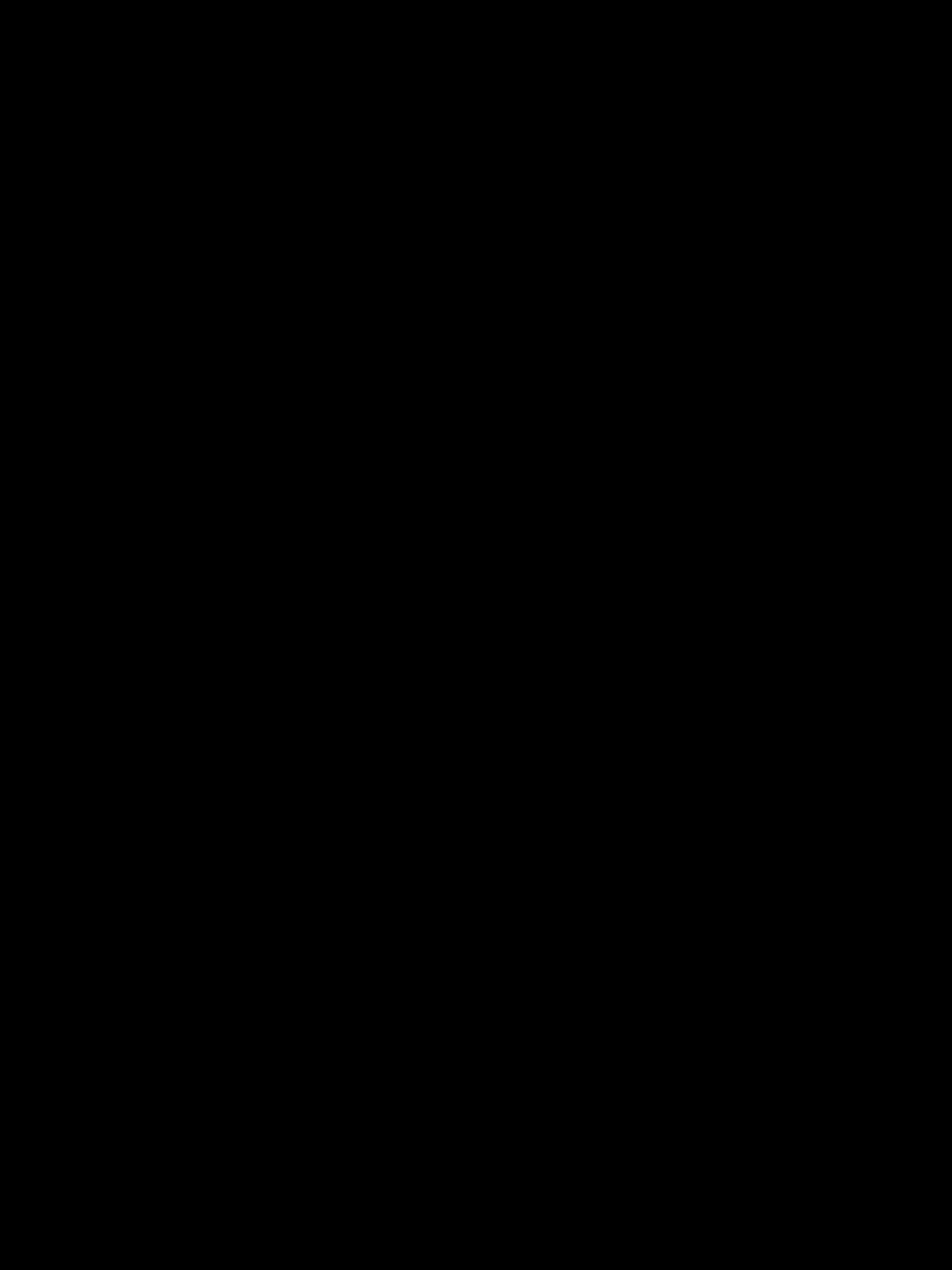

Supplement: S5 Fig — De novo motif analysis of p53 peaks in different chromatin states. The most enriched motifs were determined for each ChromHMM state by MEME. (A) Sequence logos for consensus p53 RE generated from 103 known p53 REs. (B) Sequence logos for the peaks located in active promoters closely matched the p53 consensus (motif 1) and the CTCF motif (motif 2). Peaks associated with down-regulated genes displayed CTCF motifs. (C) Several motifs recovered from peaks located in enhancers each resembled variations on the p53 consensus. (D) Sequence logos for the peaks located in heterochromatin closely matched the p53 consensus. (E) The GADD45A p53 binding site is in an open region that is well conserved with mouse. (F) In contrast the BCL2A1 peak is>20 kb upstream, shows no conservation with mouse, and is located in heterochromatin. The BCL2A1 p53RE (F) has a very high PWM, displays higher occupancy and matches the p53RE consensus better than GADD45A p53RE (E). (TIF) [file pgen.1004885.s005.tif]

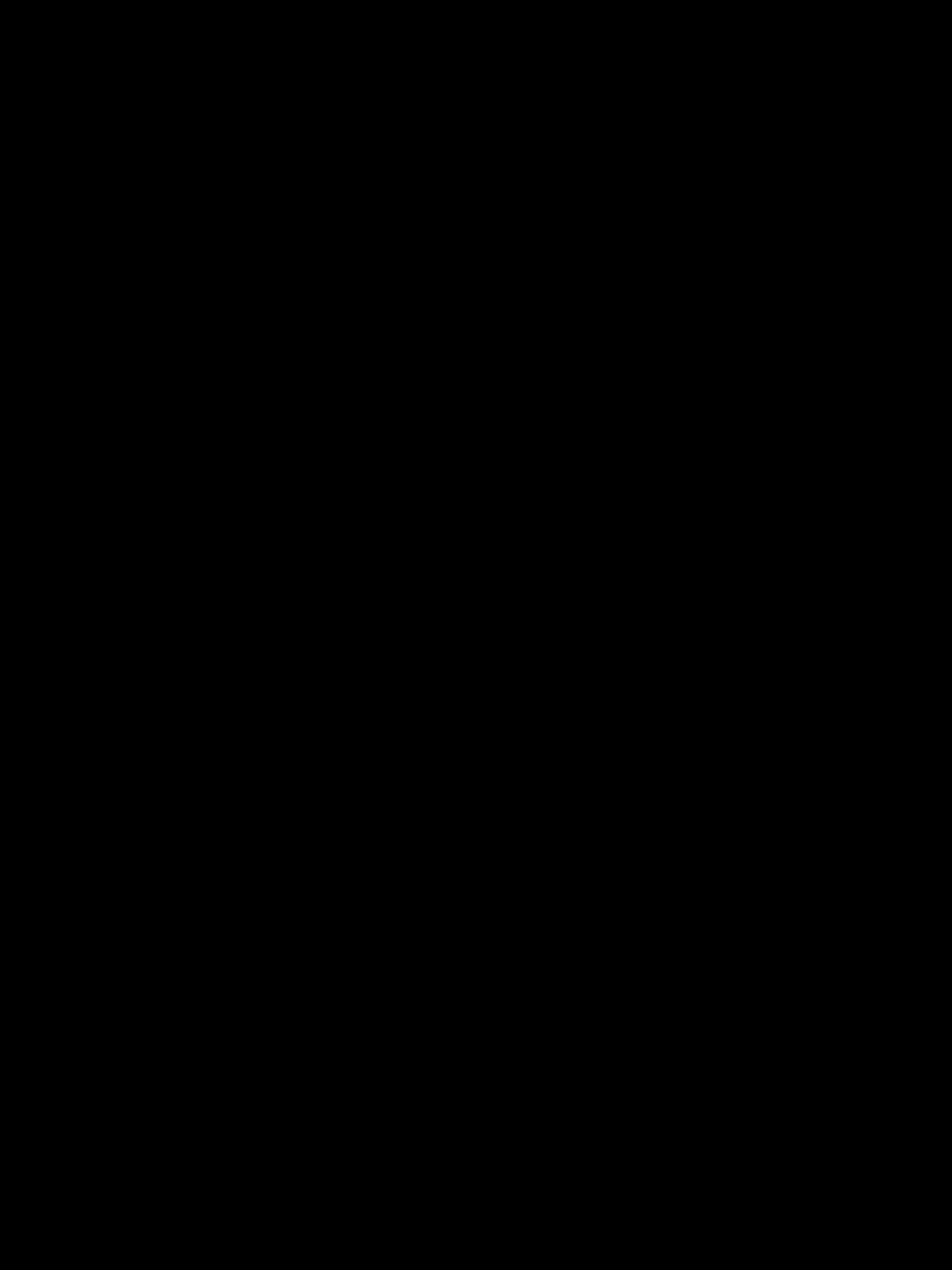

Supplement: S6 Fig — Sequence content (PWM) as organizing principle of genomic occupancy. (A) Correlation between the computed PWM and the experimentally verified binding PWM after grouping genes by PWM score deciles. (B) Left Panel, comparison of mean length of RE spacers after stratifying genes by their chromatin states at REs. Right Panel, frequency of individual spacer lengths among chromatin states. (C) Comparison of the sum of PWMs of all p53REs under each p53 peak by chromatin state at peaks. Weaker p53REs that are located in active promoters also have larger spacers. (D) Genes were grouped by ChromHMM at p53 peaks, the mean p53RE PWM score and spacer length of p53REs was computed and regressed. In vitro validation of p53 binding to 29 p53REs shown to be occupied in this experiment was reported in Noureddine et. al [31]. (TIF) [file pgen.1004885.s006.tif]

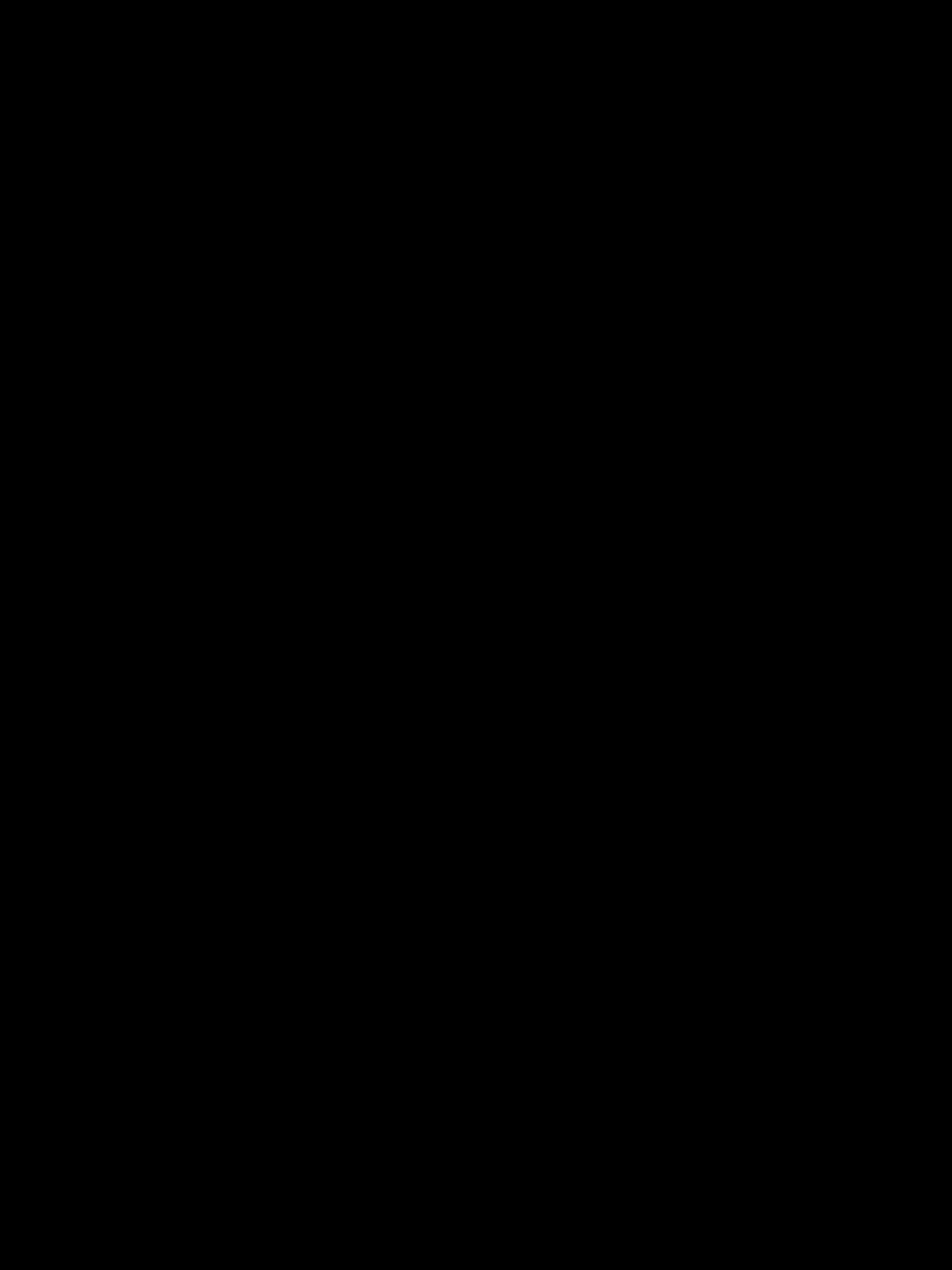

Supplement: S7 Fig — Sequence content (PWM) in relationship to p53 binding and chromatin accessibility in MCF7 cells. (A) p53 peaks in 5-FU-treated MCF7 cells are grouped based on PWM scores of REs into 10 deciles from low-to high; no RE peaks are in decile D1. (B) Linear relationship between PWM score and peak density is observed for p53 binding in MCF7 cells. (C) Inverse relationship between DHS and peak density was observed in MCF7 cells after stratifying genes by PWM deciles. Data from Nikulenkov et al.[8]. (TIF) [file pgen.1004885.s007.tif]

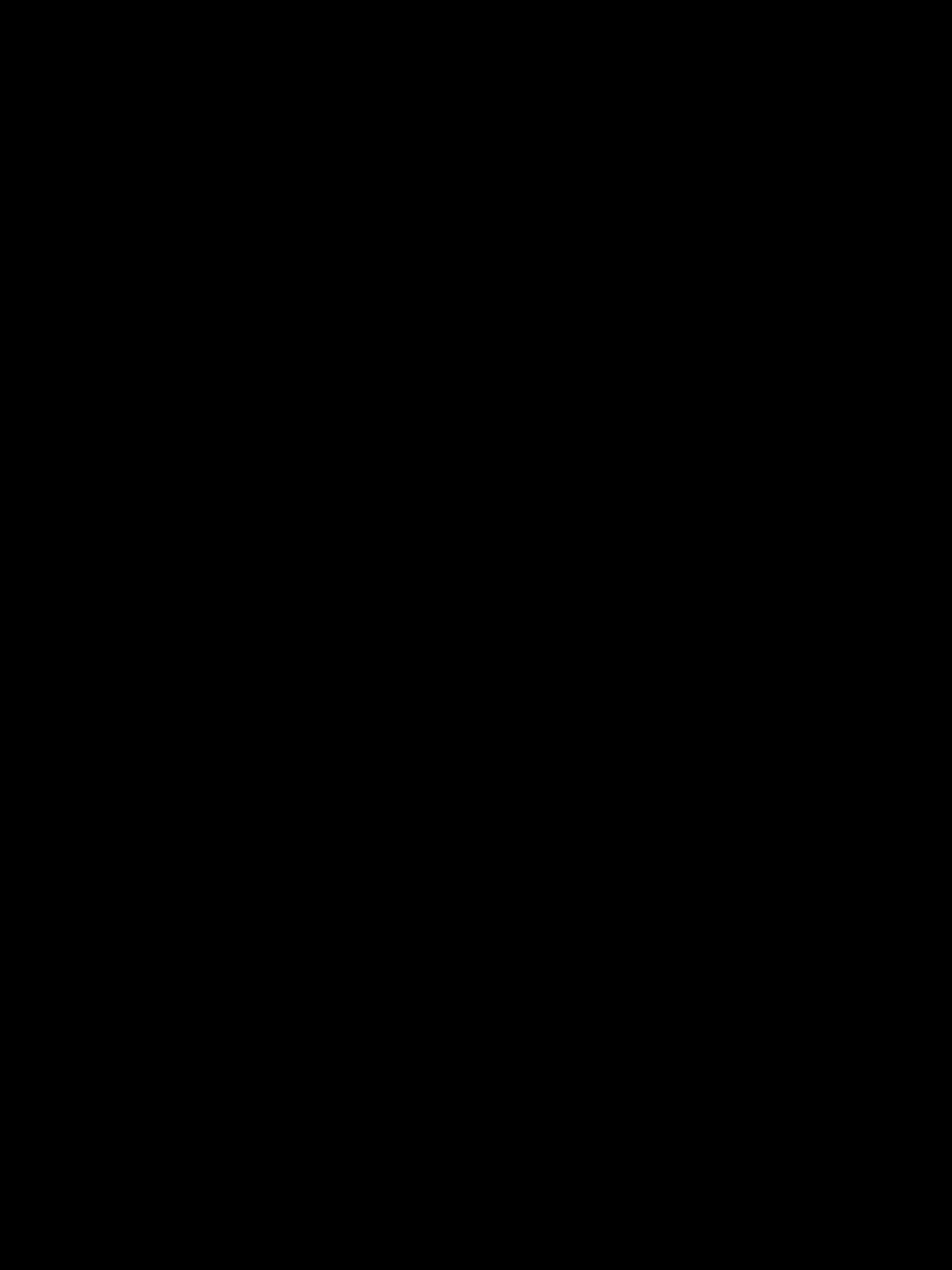

Supplement: S8 Fig — Relationships between p53 peak density, RE conservation score and PWM scores for repeats family-associated p53 RE. (A) Relationship between patterns of peak density and evolutionary conservation (PhyloP). (B) Counts of p53REs embedded in representative families of transposon elements. (C) The percentage of repeats-associated p53RE in each chromatin state. (D) Regression of p53 peak density and PWM scores for each family of repeat elements. (TIF) [file pgen.1004885.s008.tif]

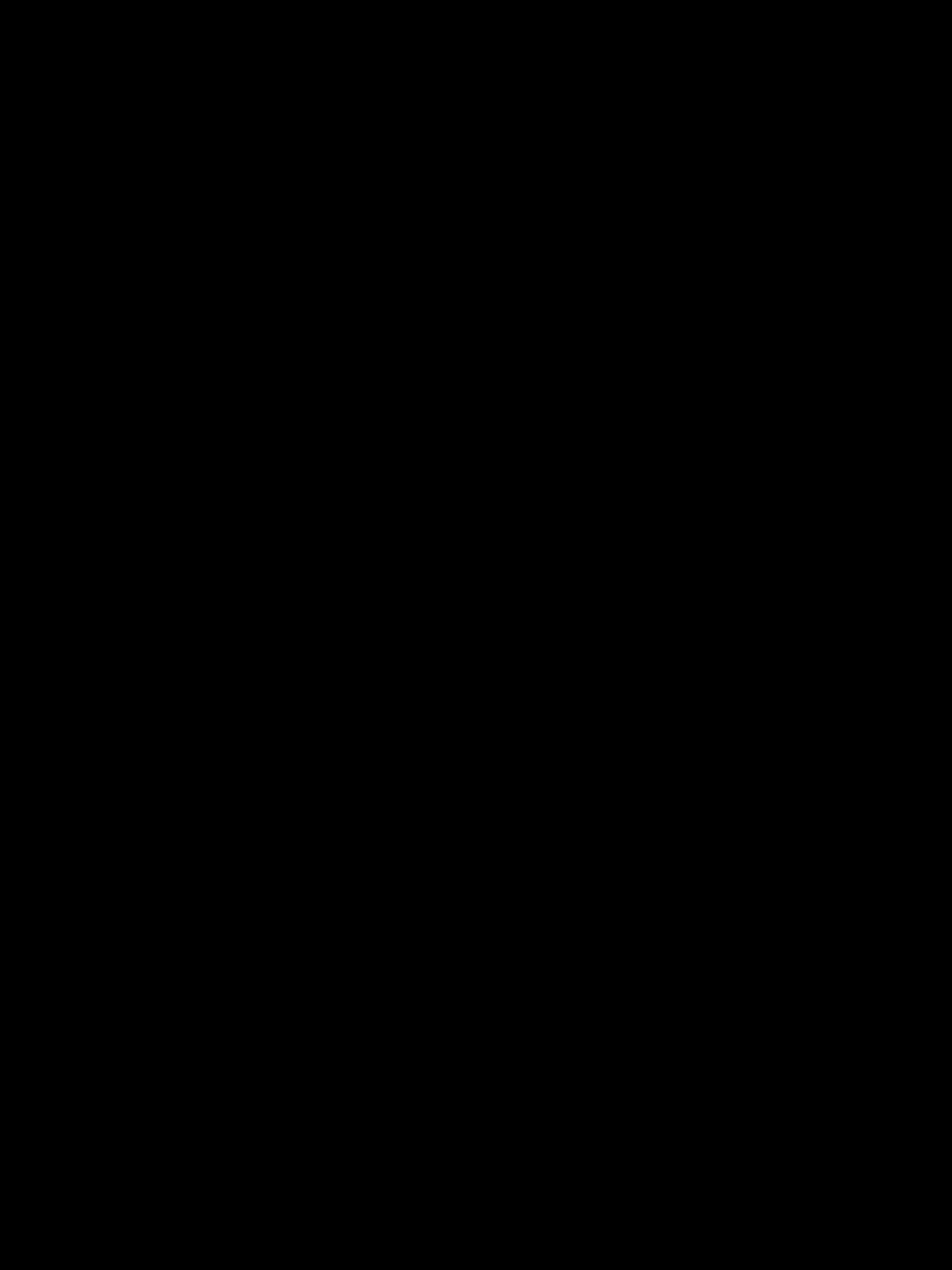

Supplement: S9 Fig — Altered DNaseI hypersensitivity at L2 transposable element associated p53 binding sites following DXR treatment. (A–B) These genes show both DNaseI change at p53RE and also H3K4me3 change at the TSS. (C–F). These genes show DNaseI change at the site of p53 binding in the TE. ChromHMM is shown in upper track, followed by DXR p53 ChIP-seq peaks, gene model, RepeatMasker tracks, PhyloP, and DNaseI hypersensitivity Seq (NT, 4 hrs DXR, 18 hrs DXR, 2 replicates each, indicated by r1, r2). (TIF) [file pgen.1004885.s009.tif]

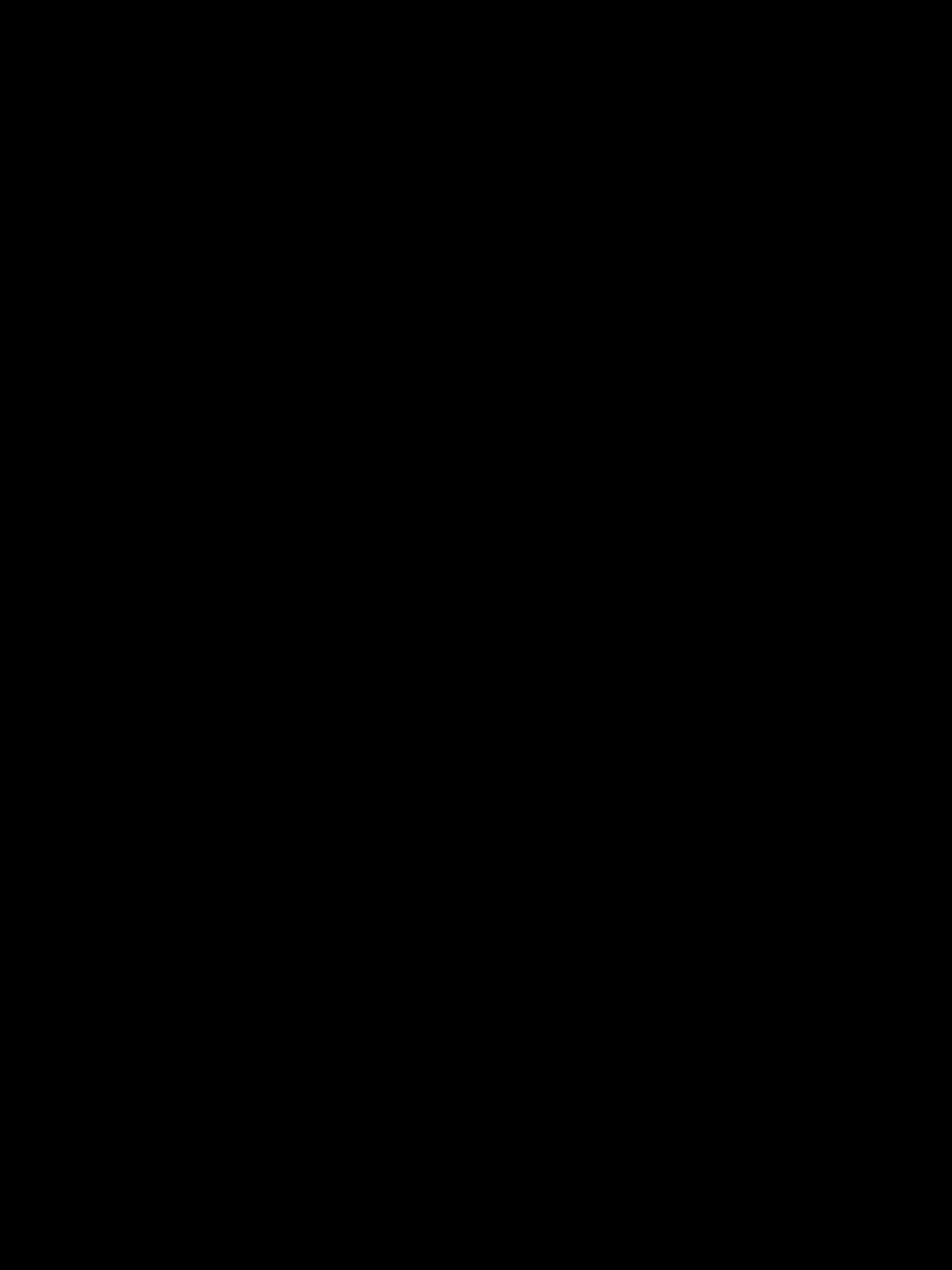

Supplement: S1 Table — Distribution of Features of High Confidence p53 Peaks and p53REs Among ChromHMM7 States. (TIF) [file pgen.1004885.s010.tif]
